# Potassium voltage-gated channel subfamily H member 2 (KCNH2) is a promising target for incretin secretagogue therapies

**DOI:** 10.1038/s41392-024-01923-z

**Published:** 2024-08-12

**Authors:** Ying-Chao Yuan, Hao Wang, Ze-Ju Jiang, Chang Liu, Qi Li, Si-Rui Zhou, Jin-Kui Yang

**Affiliations:** 1 Beijing Key Laboratory of Diabetes Research and Care, Department of Endocrinology and Metabolism, Beijing Diabetes Institute, Beijing Tongren Hospital, Capital Medical University, Beijing, 100730 China; 2 Laboratory for Clinical Medicine, Capital Medical University, Beijing, 100069 China; 3 Subcenter of State Key Laboratory of Kidney Disease, Beijing Tongren Hospital, Capital Medical University, Beijing, 100730 China

**Keywords:** Molecular biology, Biophysics, Endocrinology

## Abstract

Derived from enteroendocrine cells (EECs), glucagon-like peptide-1 (GLP-1) and glucose-dependent insulinotropic peptide (GIP) are pivotal incretin hormones crucial for blood glucose regulation. Medications of GLP-1 analogs and GLP-1 receptor activators are extensively used in the treatment of type 2 diabetes (T2D) and obesity. However, there are currently no agents to stimulate endogenous incretin secretion. Here, we find the pivotal role of KCNH2 potassium channels in the regulation of incretin secretion. Co-localization of KCNH2 with incretin-secreting EECs in the intestinal epithelium of rodents highlights its significance. Gut epithelial cell-specific KCNH2 knockout in mice improves glucose tolerance and increases oral glucose-triggered GLP-1 and GIP secretion, particularly GIP. Furthermore, KCNH2-deficient primary intestinal epithelial cells exhibit heightened incretin, especially GIP secretion upon nutrient stimulation. Mechanistically, KCNH2 knockdown in EECs leads to reduced K^+^ currents, prolonged action potential duration, and elevated intracellular calcium levels. Finally, we found that dofetilide, a KCNH2-specific inhibitor, could promote incretin secretion in enteroendocrine STC-1 cells in vitro and in hyperglycemic mice in vivo. These findings elucidate, for the first time, the mechanism and application of KCNH2 in regulating incretin secretion by EECs. Given the therapeutic promise of GLP-1 and GIP in diabetes and obesity management, this study advances our understanding of incretin regulation, paving the way for potential incretin secretagogue therapies in the treatment of diabetes and obesity.

## Introduction

Glucagon-like peptide-1 (GLP-1) and glucose-dependent insulinotropic peptide (GIP), originating from enteroendocrine L cells and K cells, respectively, play pivotal roles as incretin hormones by enhancing glucose-dependent insulin secretion from pancreatic islet β-cells.^1^ While K cells reside in the duodenum, L cells are situated in the ileum and colon, their apical surfaces directly exposed to the intestinal lumen, enabling them to sense luminal nutrients and release incretins.^2^ The incretin effect, responsible for 50–70% of normal insulin release post-glucose intake, underscores the significance of GLP-1 and GIP.^3,4^ Reduced GLP-1 and GIP levels are observed in individuals with type 2 diabetes (T2D) and glucose intolerance, and exogenous GLP-1 supplementation has proven effective in promoting insulin secretion in T2D patients.^5^ The development of GLP-1 receptor agonists (GLP-1RA) for T2D and obesity has shown cardiorenal benefits.^6^ Ongoing trials are exploring their efficacy for metabolic liver disease, peripheral artery disease, Parkinson’s, and Alzheimer’s. The success of GLP-1-based drugs has led to new molecules with unique profiles, like tirzepatide, a GIP-GLP-1 receptor coagonist. Investigational drugs such as maritide block GIP and activate GLP-1 receptors, while retatrutide and survodutide target both glucagon and GLP-1 receptors.^7,8^ However, these therapies do not target endogenous GLP-1 production, as the molecular mechanisms underlying GLP-1 secretion are not yet fully understood.

To utilize natural endogenous incretin secretion in therapy, understanding the molecular mechanisms driving hormone release from enteroendocrine cells (EECs) is essential for mobilizing endogenous incretins. Although the intracellular mechanisms driving incretin secretion in intestinal endocrine cells remain poorly understood, they may be similar to the electrical activity-regulated mechanisms in pancreatic β-cells, suggesting the presence of conserved secretory pathways.^9,10^ In enteroendocrine cells, glucose uptake, mediated by glucose transporters, induces cell membrane depolarization and calcium influx, triggering incretin release.^11,12^ The potassium channels regulating hormone secretion involves the voltage-gated potassium channel (Kv) family (repolarization), alongside ATP-sensitive potassium ion channels (K_ATP_) (depolarization). Kv channels, critical for action potential repolarization in excitable tissues, facilitate potassium efflux upon activation, resulting in membrane repolarization or hyperpolarization.^13,14^ Dysfunction or blockade of Kv channels can extend action potential duration (APD) by impeding potassium ion efflux during repolarization, thereby regulating hormone secretion.^15^

KCNH2, also known as Kv11.1 or hERG1, represents a prominent member of the Kv channel family.^16^ Expressed in various tissues, including intestinal K and L cells, KCNH2’s association with hypoglycemia as a side effect of its blockers suggests a potential role in regulating blood glucose homeostasis.^17,18^ Despite this, the precise involvement of KCNH2 in the regulation of incretin hormone secretion in enteroendocrine cells remains elusive.

This study explores the role and mechanism of KCNH2 using both in vivo mouse models and an in vitro enteroendocrine cell line. Our investigation reveals the expression of KCNH2 in GIP-producing K cells and GLP-1-producing L cells in mice. Notably, specific knockout of gut epithelial cells KCNH2 in mice enhances incretin levels, particularly GIP, concurrently improving glucose tolerance. KCNH2 knockdown diminishes Kv currents, prolongs repolarization, extends APD, and increases calcium ion flow. The KCNH2-specific inhibitor dofetilide significantly promotes incretin secretion after glucose load in enteroendocrine STC-1 cells in vitro and in hyperglycemic mice in vivo. Our research provides a distinctive mechanism facilitating incretin secretion from enteroendocrine cells and offers new strategies to enhance endogenous incretin secretion for the treatment of metabolic diseases such as diabetes and obesity.

## Result

### KCNH2 expression in incretin-producing enteroendocrine cells

To elucidate the expression levels of KCNH2 in different intestinal cells, we analyzed mouse intestinal epithelial cells using a publicly available single-cell RNA sequencing (scRNA-seq) dataset.^19^ We analyzed the expression levels of KCNH2 in five types of intestinal epithelial cells (enteroendocrine, enterocytes, paneth, tuft, and goblet cells). We found that KCNH2 was expressed in all five classes of intestinal epithelial cells, with the highest expression in enteroendocrine cells, followed by goblet, tuft, enterocytes, and paneth cells (Supplementary Fig. 1a–c). Since our study focused on the relationship between gut hormones and KCNH2, we further analyzed the mouse EECs scRNA-seq dataset^20^ and subclustered them into L cells, K cells, I cells, enterochromaffin cells (EC), X cells, D cells, N cells, and progenitor cells based on cell markers. KCNH2 was expressed in all EECs and the expression in K cells was higher than in L cells (Supplementary Fig. 1d–f).

**Fig. 1:**
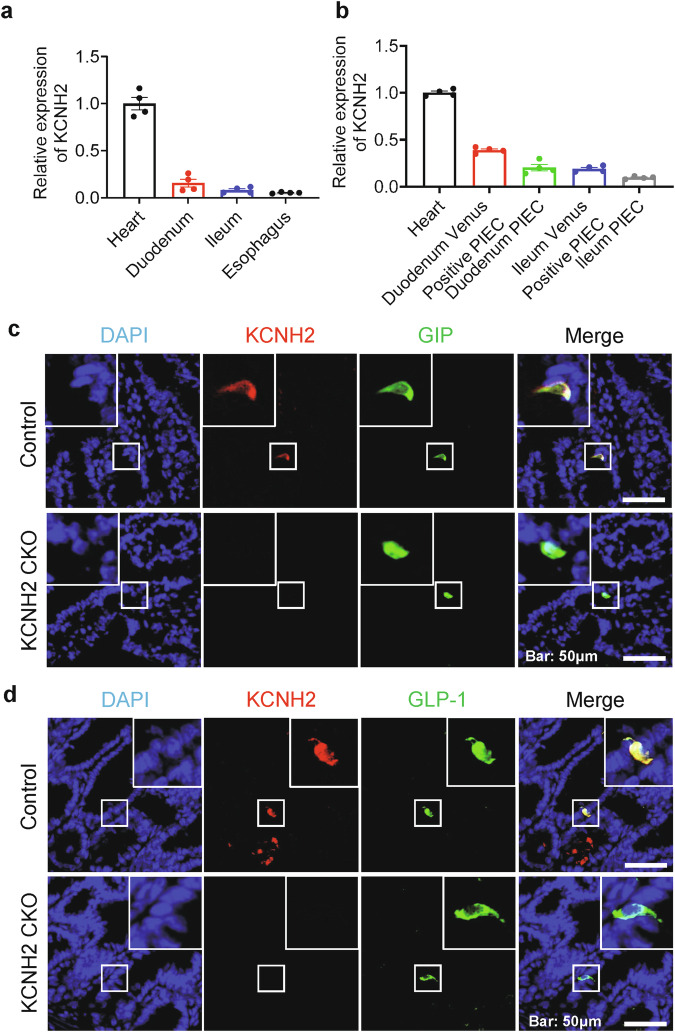
Expression of KCNH2 in incretin-producing enteroendocrine cells. **a** Relative KCNH2 mRNA expression assessed by qRT-PCR in murine heart, intestinal tissues, and esophagus (*n* = 4). **b** Relative KCNH2 mRNA expression assessed by qRT-PCR in murine heart, duodenum Venus-positive primary intestinal epithelial cells (PIEC) and ileum Venus-positive PIEC, PIEC from the duodenum (top 10 cm of the small intestine) and ileum (bottom 10 cm of the small intestine) (*n* = 4). Gene expression was quantified as 2^−ΔΔct^, with 36B4 as an internal control. **c** Immunofluorescence analysis depicted the localization of KCNH2 and GIP in the duodenum of control and KCNH2 gut epithelial cell-conditional knockout mice (CKO) mice, with a scale bar representing 50 μm. **d** Immunofluorescence staining revealed the localization of KCNH2 and GLP-1 in the ileum of control and KCNH2 CKO mice, also with a scale bar of 50 μm

Next, we examined the expression of KCNH2 in the murine small intestine, with a specific focus on incretin production. Given that KCNH2 is highly expressed in the heart, we used it as a positive control, whereas we used esophagus as a negative control because of its lower expression of KCNH2.^21^ We conducted qRT-PCR analysis and found that the expression of KCNH2 was higher in the duodenum (the top 10 cm of the small intestine) than in the ileum (the bottom 10 cm of the small intestine) (Fig. 1a). Subsequently, we further analyzed expression of KCNH2 in mouse duodenal and ileal primary epithelial cells as well as GIP-producing primary K and GLP-1-producing L cells. We used GCG-Venus transgenic mice for isolating Venus-positive primary intestinal epithelial cells (PIEC) by FACS sorting. Although K cells have traditionally been considered a distinct subset of enteroendocrine cells, there are reports in the literature that subpopulations of K cells can stain for both GLP-1 and GIP, and primary L cells collected from the proximal small intestine also express GIP.^22,23^ Our results showed that mRNA expression level of GIP in duodenum Venus-positive PIEC was 30 times higher than in duodenum Venus-negative PIEC (Supplementary Fig. 2a). In addition, mRNA expression level of proglucagon in ileum Venus-positive PIEC was 146 times higher than in ileum Venus-negative PIEC (Supplementary Fig. 2b). Therefore, we considered that Venus-positive cells isolated from the duodenum could partially represent K cells, and ileum Venus-positive PIEC could represent L cells. The expression level of KCNH2 in ileum Venus-positive PIEC is about 19% of heart, however, the expression level of KCNH2 in duodenum Venus-positive PIEC is about 38% of the heart, which is twice than in ileum Venus-positive PIEC (Fig. 1b).

Thereafter, we assessed the protein expression levels of KCNH2 in murine duodenal and ileal primary epithelial cells through Western Blotting experiments. The results showed that KCNH2 expression in duodenal epithelial cells was approximately double that observed in ileal epithelial cells, consistent with the mRNA expression data (Supplementary Fig. 2c, d). Furthermore, immunofluorescence results indicated that KCNH2 colocalized with GIP in the duodenum and GLP-1 in the ileum. This co-localization suggested the presence of KCNH2 in both GIP-produced K cells and GLP-1-produced L cells (Fig. 1c, d). Although our data only showed localization of KCNH2 in K cells and L cells since our purpose is on incretins, it is important to note that its expression is not exclusive to these two EECs but also to other EECs as shown in our scRNAseq data. These findings suggested that KCNH2 was expressed in K cells and L cells, and the expression of KCNH2 in K cells was higher than in L cells.

### Generation of KCNH2 intestine-conditional knockout mice

To examine whether intestinal KCNH2 plays a role in metabolism, we generated KCNH2 gut epithelial cell-conditional knockout mice (KCNH2^fl/fl^; Vil1-iCre, referred to as CKO hereafter) and control mice (KCNH2^fl/fl^) (Supplementary Fig. 3).^24^ Meanwhile, we found that KCNH2 expression was significantly depleted in the duodenum and ileum of CKO mice, whereas knockout of KCNH2 did not affect the basal level/distribution of GIP/GLP-1 (Fig. 1c, d).

Firstly, we closely monitored the body weight changes of mice weekly under different dietary conditions. We found that the body weights of KCNH2 CKO mice was not changed compared with control mice under a regular-chow diet. However, the body weights of KCNH2 CKO mice began to increase slowly after application of the high-fat diet and became significantly lower than those of control mice (Supplementary Fig. 4a). In addition to body weight, we also monitored food intake and water consumption, and found that the quantity of food intake of CKO mice was lower than that of the control group, while water consumption was not changed between the two groups (Supplementary Fig. 4b, c).

We also evaluated various parameters in adult mice (12 weeks old) and observed that CKO mice exhibited body length, and small intestine length (normalized to body length) comparable to those of the control group (Supplementary Fig. 4f–h). Gross intestinal morphology, intestinal villus height, and crypt depth of adult CKO mice remained consistent with those of the control (Supplementary Fig. 4d, i, j). Immunostaining for insulin indicated that the morphology, size, and number of pancreatic islets in CKO mice were not altered when compared to control mice (Supplementary Fig. 4e, k, l). These results indicated that intestinal KCNH2 knockout lead to reduced food intake and weight gain in mice on a high-fat diet condition, but had no effect on the growth of small intestine and islets.

### KCNH2 deficiency decreases glucose levels via incretins

**Fig. 2:**
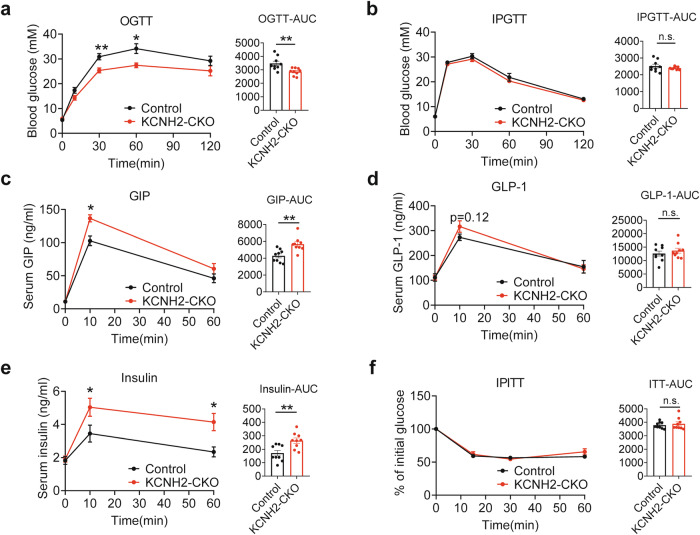
KCNH2 deficiency enhances insulin and incretin secretion in response to glucose in vivo. **a** Blood glucose levels and area under the curve (AUC) during an oral glucose tolerance test (OGTT, 5 g/kg) for mice (high-fat diet (HFD)-fed for 6–8 weeks, started at week 8) (Control, *n* = 9; CKO, *n* = 9). **b** Blood glucose levels and AUC during an intraperitoneal glucose tolerance test (IPGTT) (2 g/kg) for mice (HFD-fed for 6–8 weeks, started at week 8) (Control, *n* = 9; CKO, *n* = 9). **c**–**e** Serum GIP (**c**), GLP-1 (**d**), and insulin levels (**e**), along with their AUC during OGTT (5 g/kg) for mice (HFD-fed for 6–8 weeks, started at week 8) (Control, *n* = 9; CKO, *n* = 9). **f** Blood glucose levels and AUC during an intraperitoneal insulin tolerance test (IPITT) (0.75 U/kg) for mice (HFD-fed for 6–8 weeks, started at week 8) (Control, *n* = 9; CKO, *n* = 9). Values are means ± SEM. Statistical significance was assessed by Student’s *t*-test. *p < 0.05; **p < 0.01; n.s. means not significant

To investigate the impact of intestinal KCNH2 on metabolism, we conducted a glucose tolerance test in CKO and control mice. Our data revealed no differences in basal glucose levels between CKO and control mice. However, the oral glucose tolerance test (OGTT) indicated improved glucose disposal in CKO mice (Fig. 2a). Interestingly, there were no differences in blood glucose levels between control and CKO mice in the intraperitoneal glucose tolerance test (IPGTT) (Fig. 2b), suggesting that intestinal KCNH2 knockout did not affect islet function.

To explore the reasons behind the improved glucose tolerance in CKO mice, we examined the incretin levels during OGTT. Upon oral glucose administration, GIP levels significantly increased in CKO mice compared to control mice (Fig. 2c), while GLP-1 levels in CKO mice exhibited a slight elevation without reaching statistical significance (Fig. 2d). Serum insulin concentration was enhanced following glucose challenge in CKO mice (Fig. 2e). No differences in glucose levels were observed in the insulin tolerance test (Fig. 2f), suggesting that intestinal KCNH2 knockout improves glucose homeostasis independently of insulin sensitivity. These findings indicate that intestine-specific KCNH2 knockout regulates systemic glucose homeostasis through increased incretin levels.

The results of single-cell sequencing indicated that KCNH2 is widely expressed in intestinal EECs, indicating that KCNH2 may be involved in other gut hormones release. We checked other serum intestinal hormone concentrations after glucose load from high-fat diet-induced KCNH2 CKO and control mice. The results showed that KCNH2 CKO mice exhibited increased levels of peptide YY (PYY) and somatostatin but normal level of cholecystokinin (CCK) concentration in serum after glucose challenge (Supplementary Fig. 5a–c).

Previous research has suggested that host intestinal ion transport affects the luminal environment and regulates the composition of the intestinal microbiota, which is associated with incretin secretion.^25,26^ Here, we explored whether the knockout of intestinal KCNH2 has an impact on the gut microbiota and, consequently, regulates incretin secretion. Nevertheless, our research showed no appreciable variations in the gut microbiota’s composition, diversity, or abundance between CKO and control mice (Supplementary Fig. 6). This suggests that intestinal KCNH2 knockout has no effect on the gut microbiota’s bacterial diversity, abundance, or composition.

### Intestinal KCNH2 deficiency does not affect incretin synthesis and degradation

**Fig. 3:**
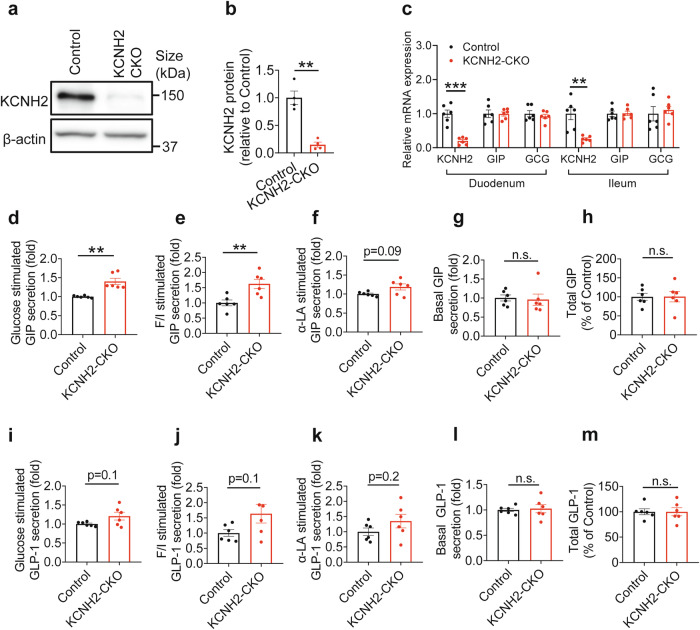
KCNH2-deficient PIEC showed enhanced incretin secretion. **a**, **b** Protein levels of KCNH2 in PIEC from Control and CKO mice assessed by immunoblotting (*n* = 3 mice per group). **c** Quantitative PCR was used to assess the relative mRNA expression of KCNH2, GIP, and GCG in the PIEC of the duodenum and ileum (*n* = 6 mice per group). 2^−ΔΔct^ was used to calculate gene expression, while 36B4 served as an internal control. **d**–**h** In vitro 10 mM glucose (**d**) or 10 mM glucose supplemented with 10 μM Forskolin plus 10 μM IBMX (**e**), 50 μM α-LA (**f**) or basal conditions (0 mM glucose), **g** induced GIP secretion and total GIP content (**h**) in duodenal PIEC of Control and CKO mice (*n* = 6 replicates per group). **i**–**m** In vitro 10 mM glucose (**i**) or 10 mM glucose supplemented with 10 μM Forskolin plus 10 μM IBMX (**j**), 50 μM α-LA (**k**) or basal conditions (0 mM glucose), **l** induced GLP-1 secretion and total GLP-1 content (**m**) in ileal PIEC of Control and CKO mice (*n* = 6 replicates per group). Values are means ± SEM. Statistical significance was assessed by Student’s *t*-test. **p < 0.01; ***p < 0.001; n.s. means not significant

The intestinal EECs include GIP-producing K cells and GLP-1-producing L cells; K cells are mostly found in the duodenum, whereas L cells are primarily found in the ileum and colon.^27,28^ Next, we further explore the role of KCNH2 in intestinal cell function. Due to the inability of individual K or L cells to survive in culture, we isolated primary intestinal epithelial cells from the duodenum (the top 10 cm of the small intestine) or ileum (the bottom 10 cm of the small intestine) of KCNH2 CKO and control mice, respectively.^11,29^ qRT-PCR and western blotting verified that mRNA and protein levels of KCNH2 were significantly reduced in both duodenal and ileal PIEC cultures from CKO mice (Fig. 3a–c).

Next, we investigated whether KCNH2 deficiency in primary intestinal epithelial cells is associated with changes in incretin synthesis and degradation. We assessed the mRNA levels of GIP and GCG in PIEC from the duodenum and ileum and found no differences between CKO and control group (Fig. 3c). Since the PCSK1 and PCSK2 processes its precursors (proglucagon in L cells and proGIP in K cells) to form GLP-1 and GIP,^30^ we analyzed the expression of PCSK1 and PCSK2 in PIEC from the duodenum and ileum of control and KCNH2 CKO mice under basal and glucose-stimulated conditions. Our results showed that mRNA levels of PCSK1 and PCSK2 in intestinal epithelial cells from control and CKO mice were not changed under each condition (Supplementary Fig. 7a–d). These results indicated that intestinal KCNH2 deficiency does not alter the expression of PCSK1 and PCSK2 in intestinal epithelial cells.

In L cells, PCSK1 processes proglucagon to form GLP-1 and GLP-2.^31^ We then explored the effects of KCNH2 knockout on glucagon and GLP-2 secretion after glucose stimulation. We conducted oral glucose stimulation in high-fat diet-induced control and CKO mice and found no significant change in glucagon and GLP-2 levels in both groups before or after glucose stimulation (Supplementary Fig. 5d, e). Then, we evaluated whether KCNH2 CKO affects intestinal glucagon and GLP-2 secretion and the content of PIEC from the mouse duodenum and ileum with or without glucose stimulation. The results showed that either secretion or content of glucagon in primary intestinal epithelial cells from KCNH2 CKO mice was not changed compared with those of control mice before or after glucose stimulation (Supplementary Fig. 7e-h). In addition, a similar result was also exhibited that secretion and content of GLP-2 in PIEC from KCNH2 CKO was also not changed (Supplementary Fig. 7i–l). These findings demonstrated that KCNH2 deficiency in intestinal epithelial cells did not affect either secretion or content of glucagon and GLP-2 before or after glucose stimulation.

Since secreted GLP-1 and GIP will be broken down with DPP-IV enzyme in circulation, we also assessed the expression of DPP-IV in Control and CKO mice with or without stimulation.^32^ We found that mRNA levels of DPP-IV in the primary intestinal epithelial cells from CKO mice were similar to those from control mice under basal or glucose stimulation conditions (Supplementary Fig. 7m, n). Subsequently, we examined the serum levels of DPP-IV before or after glucose stimulation in control and KCNH2 CKO mice and found there was no difference between these two groups (Supplementary Fig. 5f). Overall, these findings suggested that intestinal KCNH2 deficiency does not affect the synthesis and degradation of incretins.

### KCNH2-deficient intestinal cells exhibit increased incretin secretion

Since intestinal KCNH2 deficiency does not affect incretin synthesis and degradation, we speculated that the effect of KCNH2 deficiency was might be involved in incretin secretion. To confirm our speculation, we first analyzed incretin secretion under stimulated conditions. Under basal conditions, we observed that KCNH2 knockout did not affect GLP-1 and GIP secretion (Fig. 3g, l). However, under glucose stimulation, PIEC from KCNH2 CKO mice exhibited increased GIP and GLP-1 secretion, with a more pronounced effect on GIP (Fig. 3d, i). We then evaluated total GIP in duodenal PIEC cultures and GLP-1 levels in ileal PIEC cultures, respectively. No differences were found in total GLP-1 and GIP levels between CKO and control mice, indicating that KCNH2 knockout does not affect GIP and GLP-1 expression in intestinal cells (Fig. 3h, m).

To assess the impact of additional stimuli, we employed 10 μM forskolin in combination with 10 μM 3-isobutyl-1-methylxanthine (IBMX) to simulate cAMP stimulation^33^ and 50 μM α-linolenic acid (α-LA) to mimic fatty acid stimulation.^34^ Stimulation with the cAMP agonists (Forskolin + IBMX) significantly enhanced GIP secretion in KCNH2 CKO PIEC and led to a slight increase in GLP-1 levels (Fig. 3e, j). Conversely, α-LA stimulation resulted in only a minor increase in GIP and GLP-1 secretion (Fig. 3f, k).

These findings indicate that the absence of KCNH2 enhances incretin secretion, especially GIP, following nutrient stimulation, without affecting incretin content.

### KCNH2 knockdown increases incretin secretion

**Fig. 4:**
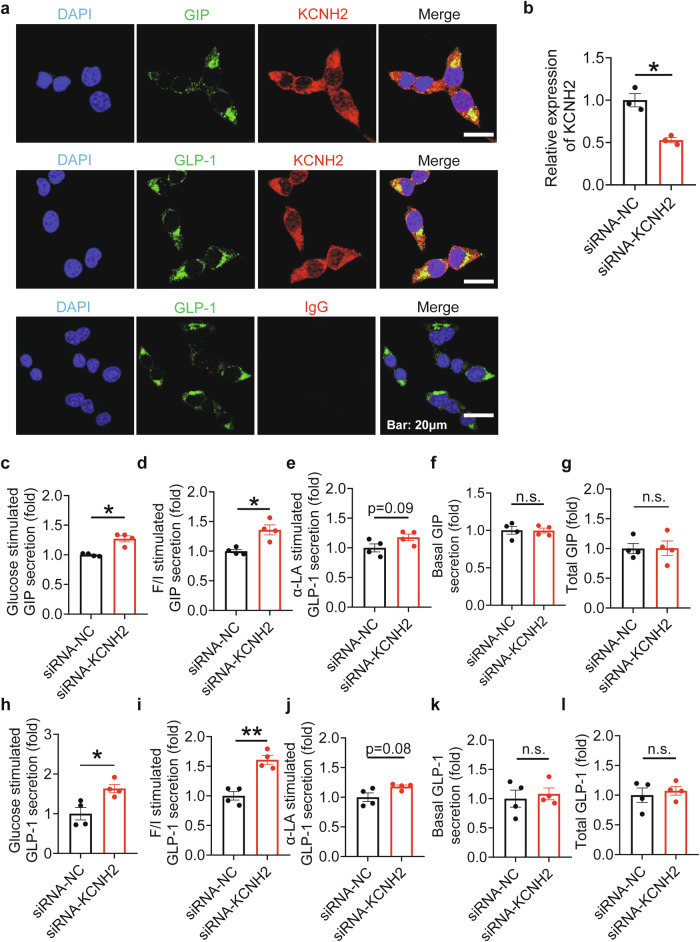
Increased incretin secretion in KCNH2-deficient enteroendocrine cells after stimulation. **a** Immunofluorescence analysis depicted the localization of KCNH2 and GIP/ GLP-1 in STC-1 cells. The scale bar shows 20 μm. At least three replications of each experiment were conducted. **b** Transfection of the enteroendocrine cell line (STC-1 cells) with either 50 nM scramble siRNA (siRNA-NC) or siRNA against KCNH2 (siRNA-KCNH2) for 48 h, assessed by qPCR (siRNA-NC, *n* = 3; siRNA-KCNH2, *n* = 3). **c**–**g** STC-1 cells were incubated in Krebs–Ringer bicarbonate buffer (KRBB) for 30 min, followed by the 10 mM glucose or 10 mM glucose supplemented with 10 μM Forskolin plus 10 μM IBMX (F/I) or 50 μM α-LA or 0 mM glucose (basal) KRBB for an additional 2 h. Glucose (**c**), F/I (**d**), α-LA (**e**), and basal conditions (**f**) induced GIP secretion and total GIP content (**g**) in siRNA-NC and siRNA-KCNH2 STC-1 cells (*n* = 4 replicates for each group). **h**, **l** Glucose (**h**), F/I (**i**), α-LA (**j**), and basal conditions (**k**) induced GLP-1 secretion and total GLP-1 content (**l**) in siRNA-NC and siRNA-KCNH2 STC-1 cells (*n* = 4 replicates for each group). Values are means ± SEM. Statistical significance was assessed by the Student’s *t*-test. *p < 0.05;**p < 0.01; n.s. means not significant

To explore the exact molecular mechanism by which KCNH2 modulates incretin, we used in vitro-cultured STC-1 cells, a mature mouse enteroendocrine cell line that secretes a variety of hormones, including GIP and GLP-1.^11^ To determine the distribution of KCNH2 on STC-1 cells, we performed immunofluorescence staining. The results demonstrated the presence of KCNH2 in STC-1 cells, where it colocalized with GIP and GLP-1 (Fig. 4a). To assess whether the knockdown of KCNH2 channels affects the incretin of STC-1 cells, we employed siRNA to suppress KCNH2 expression in these cells. A substantial decrease in KCNH2 expression relative to the control group (siRNA-NC) was verified by qRT-PCR (Fig. 4b).

Furthermore, we evaluated the effect of KCNH2 deficiency on incretin secretion in STC-1 cells. Depletion of KCNH2 expression did not affect the total GIP and GLP-1 content (Fig. 4g, l). Subsequently, we stimulated STC-1 cells to observe the effect of KCNH2 knockdown on incretin secretion. Under basal conditions, incretin secretion remained unchanged between the two groups (Fig. 4f, k). However, after stimulation with glucose or forskolin plus IBMX, KCNH2 depletion significantly increased GIP and GLP-1 secretion, while incretin secretion was slightly elevated by α-LA stimulation (Fig. 4c–e, h–j).

### KCNH2 knockdown inhibits Kv currents, prolongs APD, and increases Ca^2+^ concentration

**Fig. 5:**
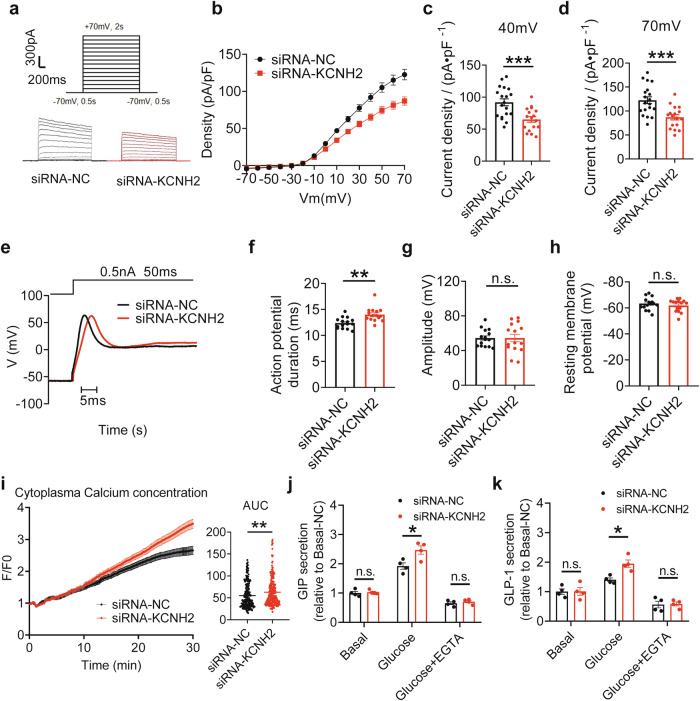
KCNH2 knockdown inhibits Kv currents, prolongs APD, and increases Ca^2+^ concentration in enteroendocrine cells. **a** Representative Kv currents of STC-1 cells treated with siRNA-NC or siRNA-KCNH2. The cells were held at a voltage of −70 mV for 2 s before being depolarized to 70 mV in steps of 10 mV. **b**–**d** Summary of the steady-state current-voltage (I-V) curves for Kv currents (**b**) and mean Kv current densities at +40 mV (**c**) and +70 mV (**d**) in STC-1 cells (siRNA-NC, *n* = 19; siRNA-KCNH2, *n* = 17). **e** Representative action potentials captured in current-clamp mode from STC-1 cells treated with siRNA-NC or siRNA-KCNH2. **f**–**h** Summary of action potential durations (**f**), amplitude (**g**), and resting membrane potential (**h**) in STC-1 cells (siRNA-NC, *n* = 15; siRNA-KCNH2, *n* = 15). **i** Intracellular calcium concentration was ascertained using 2 μM Fluo4-AM. For a total of 31 min, readings were obtained every 5 s, with 60 s recorded before and 30 min following stimulation with 10 mM glucose and 10 μM forskolin plus IBMX. The fluorescence change ratio (F/F0) was obtained for STC-1 cells treated with scramble siRNA and KCNH2 siRNA. The summary of area under curve (AUC) of STC-1 cells (siRNA-NC, *n* = 232; siRNA-KCNH2, *n* = 244). **j**, **k** STC-1 cells were incubated in Krebs–Ringer bicarbonate buffer (KRBB) or KRBB supplemented with 10 mM EGTA for 30 min, followed by the 10 mM glucose or 10 mM glucose supplemented with 10 mM EGTA or 0 mM glucose (basal) KRBB for an additional 2 h. GIP secretion (**j**) and GLP-1 secretion (**k**) in siRNA-NC and siRNA-KCNH2 STC-1 cells (*n* = 4 replicates for each group). Values are means ± SEM. Statistical significance was assessed by Student’s *t*-test. **p < 0.01; ***p < 0.001; n.s. means not significant

Since our study has demonstrated that KCNH2 plays a role in incretin secretion, we employed STC-1 cells, which secrete both GIP and GLP-1, to investigate the electrophysiological mechanism.^35,36^ To assess whether the knockdown of KCNH2 channels affects the electrophysiological activity of STC-1 cells, we employed siRNA to suppress KCNH2 expression in these cells. Kv channels are essential for action potential repolarization in excitable cells, and their blockade or loss of function can lead to a reduction in potassium ion outflow during repolarization, consequently prolonging the APD.^37^ Since KCNH2 is a member of the voltage-gated potassium channel family, we recorded whole-cell total Kv currents (Fig. 5a). Current densities were calculated as a representation of the overall channel function. Our analysis revealed that Kv currents in STC-1 cells treated with KCNH2 siRNA was significantly lower than in control cells, with a more pronounced reduction at higher voltages (Fig. 5b–d).

To determine the impact of KCNH2 knockdown on APD in STC-1 cells, we induced electrical action potentials in current-clamp mode. The findings showed that action potential repolarization was prolonged in KCNH2 siRNA-treated cells in comparison to control cells (Fig. 5e, f). However, action potential amplitude and resting membrane potential did not significantly differ between the two groups (Fig. 5g, h).

A previous study has indicated that blockage of KCNH2 channels can elevate intracellular calcium concentration ([Ca^2+^]i) in both pancreatic islet α and β cells.^38^ To further investigate the function of KCNH2 channels in calcium homeostasis, we inhibited KCNH2 expression in STC-1 cells using siRNA and measured the response of [Ca^2+^]i to high glucose and stimulators, including forskolin plus IBMX. Compared to the control (siRNA-NC), we observed an increased intracellular calcium concentration in KCNH2 siRNA-treated STC-1 cells after stimulation (Fig. 5i).

To better understand the relationship between calcium homeostasis and the release of incretins, we used EGTA, a calcium chelator, to check incretin secretion with glucose stimulation in STC-1 cells.^39^ The result showed that KCNH2-downregulated STC-1 exhibited significantly increased GIP and GLP-1 secretion after stimulation with glucose. However, elevated secretion of GIP and GLP-1 was diminished after adding EGTA in both KCNH2-downregulated and control STC-1 cells (Fig. 5j, k). These findings indicated that the glucose-stimulated incretin secretion in KCNH2-downregulated or control STC-1 cells is calcium-dependent.

In summary, KCNH2 channels regulate repolarizing Kv currents, and their reduction leads to prolonged APD and enhances [Ca^2+^]i in glucose-stimulated STC-1 cells.

### Dofetilide promotes incretin secretion in mice in vivo and in EECs in vitro

**Fig. 6:**
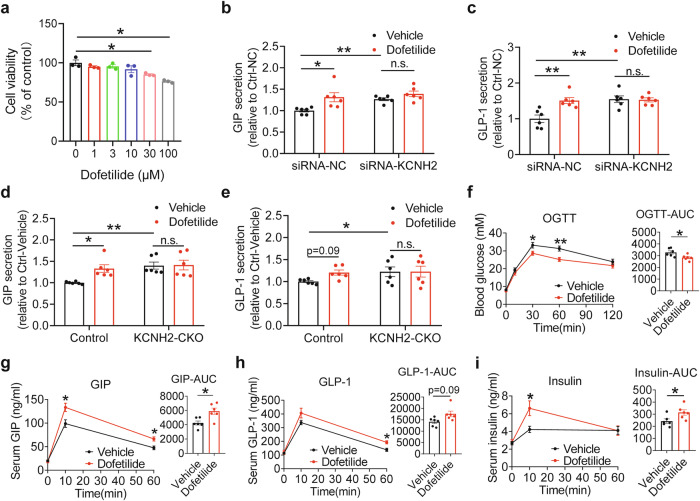
Dofetilide promotes incretin secretion in mice in vivo and in EECs in vitro. **a** Viability of STC-1 cells incubated with various concentrations of dofetilide for 2 h. (*n* = 3 replicates for each group). **b**, **c** The enteroendocrine cell line (STC-1 cells) were transfected for 48 h with either 50 nM scramble siRNA (siRNA-NC) or siRNA against KCNH2 (siRNA-KCNH2). STC-1 cells were incubated in Krebs–Ringer bicarbonate buffer (KRBB) for 30 min followed by 10 mM glucose KRBB plus vehicle or 10 mM glucose KRBB supplemented with 10 μM dofetilide for an additional 2 h. Glucose-induced GIP secretion (**b**) and GLP-1 secretion (**c**) (*n* = 6 replicates per group). **d**, **e** PIEC were cultured in KRBB for 30 min and then treated with 10 mM glucose KRBB plus vehicle or 10 mM glucose KRBB supplemented with 10 μM dofetilide for an additional 2 h. Glucose-induced GIP secretion (**d**) and GLP-1 secretion (**e**) (*n* = 6 replicates for each group). **f**–**i** HFD-fed (started at week 8 for 6–8 weeks) C57BL/6J were orally administered 5 mg/kg dofetilide or vehicle before being loaded with 5 g/kg glucose for the OGTT. Blood glucose levels and AUC (**f**), serum GIP levels and AUC (**g**), serum GLP-1 levels and AUC (**h**), and serum insulin levels and AUC (**i**) (Vehicle, *n* = 6; Dofetilide, *n* = 6). The values are presented as means ± SEM. The statistical significance of differences between means was assessed by the Student’s *t*-test. *p < 0.05; **p < 0.01; n.s. means not significant

Our study found that knockout or knockdown of KCNH2 can promote the secretion of incretins. Therefore, we used the KCNH2-specific inhibitor dofetilide to explore its effects on incretin secretion.^40^ Dofetilide is a relatively new class III antiarrhythmic medication that specifically inhibits the rapid component of the delayed rectifier potassium current in cardiac ion channels. It has demonstrated remarkable efficacy in converting atrial fibrillation or flutter to sinus rhythm.^41,42^ Firstly, dofetilide was non-toxic to cell viability at concentrations of 10 μm or less (Fig. 6a). Secondly, we found that dofetilide significantly increased glucose-induced GLP-1 and GIP secretion in control STC-1 cells. However, it had no significant promoting effect on KCNH2-knockdown STC-1 cells (Fig. 6b, c). Meanwhile, we used the KCNH2-specific inhibitor dofetilide for detecting intracellular calcium concentration in STC-1 cells. The results revealed that dofetilide enhanced the intracellular calcium levels in STC-1 cells after glucose stimulation, suggesting that blockade of KCNH2 raises the intracellular calcium concentration, which further promotes incretin secretion from STC-1 cells (Supplementary Fig. 8).

Next, we extracted primary intestinal epithelial cells from the duodenum and ileum of mice and found that dofetilide increased GIP secretion in duodenal epithelial cells by 30% and increased GLP-1 secretion in ileal epithelial cells by 20%. However, dofetilide had no significant effect on intestinal epithelial cells from KCNH2 CKO mice, neither promoting GIP secretion nor GLP-1 secretion (Fig. 6d, e).

To better investigate the promotional effect of dofetilide on incretin, we administered dofetilide or vehicle to C57BL/6 J mice with high-fat diet (HFD)-induced hyperglycemia. Following oral glucose challenge, dofetilide markedly lowered blood glucose levels in HFD-fed mice and increased GLP-1, GIP, and insulin levels (Fig. 6f–i). These results suggest that dofetilide increases the intracellular calcium concentration by blocking KCNH2, thereby increasing incretin secretion and improving glucose homeostasis.

## Discussion

**Fig. 7:**
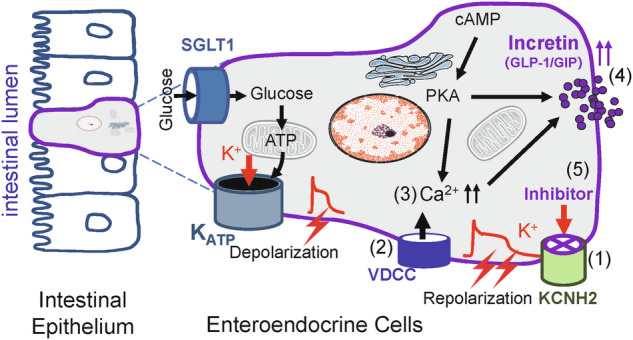
Proposed mechanisms by how dysfunction of KCNH2 channel enhances incretin secretion. In enteroendocrine cells, glucose enters the cell via the apical glucose transporter (SGLT1), which produces ATP, closes the KATP channel, depolarizing the cell membrane and allowing calcium influx, thereby prompting the release of incretin. In addition to this, we found a unique role for KCNH2 in incretin. (1) KCNH2 deficiency led to a reduction in Kv currents and prolonged repolarization in enteroendocrine cells. (2) Prolonged repolarization causes an increase in Ca^2+^ influx (3) Resulting in the accumulation of intracellular Ca^2+^. (4) Higher intracellular Ca^2+^ concentration triggers more incretin secretion. (5) KCNH2-specific inhibitor--dofetilide causes a rise in the intracellular calcium concentration by inhibiting KCNH2, thereby increasing incretin secretion. Therefore, KCNH2 has an important potential as a promising novel target for the treatment of obesity and diabetes

GIP and GLP-1 are incretins secreted within minutes of nutrient intake in the gastrointestinal tract, which enhance glucose-stimulated insulin secretion.^4,27,31^ The cellular mechanisms that control the endogenous secretion of incretins remain poorly understood. This study elucidates for the first time the role and mechanisms of the KCNH2 potassium channel in regulating the secretion of incretin hormones by enteroendocrine cells. KCNH2 deficiency reduces Kv currents, prolonging repolarization in enteroendocrine cells. This prolonged repolarization increases Ca^2+^ influx, elevating intracellular Ca^2+^ levels and triggering increased incretin secretion. Finally, the KCNH2-specific inhibitor dofetilide promotes incretin secretion by blocking KCNH2, leading to an increase in intracellular calcium concentration, which further enhances insulin secretion (Fig. 7). These findings underscore the potential of KCNH2 as a promising and novel target for the treatment of obesity and diabetes.

Understanding the functionality of EECs and the molecular mechanisms governing incretin secretion is pivotal for leveraging endogenous incretin reserves in therapeutic strategies. Glucose enters EECs via sodium/glucose cotransporter 1 (SGLT1), initiating ATP production and leading to membrane depolarization. Subsequently, calcium ions flow in through voltage-gated calcium channels, elevating intracellular calcium levels and triggering incretin secretion, which is similar to the mechanism of insulin secretion by pancreatic β-cells.^11,43–45^ Our research reveals that in addition to K_ATP_ and calcium channels, Kv channels play a crucial role in incretin secretion in EECs. KCNH2 knockdown in EECs significantly reduces Kv currents, resulting in prolonged action potential duration inducing increased intracellular calcium concentration to enhance incretin secretion. The addition of a calcium chelator abolishes the KCNH2-mediated enhancement of incretin secretion, indicating that glucose-stimulated incretin secretion in KCNH2-downregulated STC-1 cells is calcium-dependent. Furthermore, our study verified that the effect of KCNH2 deletion in enteroendocrine cells on incretin secretion could not be attributed to altered levels of incretin expression, synthesis, and degradation, as the content of GIP and GLP-1, and the expression of PCSK1/PCSK2 and DPP-4 were similar in CKO and control mice. These findings suggest that KCNH2 plays a role in action potential repolarization and exerts regulatory control over incretin secretion in EECs.

Our research underscores the distinct regulatory impact of KCNH2 on incretin secretion, particularly favoring GIP over GLP-1, as evident from both in vivo and in vitro experiments involving CKO mice. We compared the expression of KCNH2 in different EECs of the intestine and found that the expression level of KCNH2 in K cells (GIP producer) was higher than in L cells (GLP-1 producer) which indicated that KCNH2 may play a more crucial role in GIP secretion from duodenal K cells than in GLP-1 secretion from ileal L cells. Similar observations of compartmentalized regulation have been reported in other studies. For instance, GPR142, a putative amino acid receptor is expressed at higher levels in the upper small intestine compared to the distal segments, and GIP secretion decrease after food intake, while GLP-1 secretion remains unaffected in GPR142-deficient mice.^46^ Although the precise mechanisms underpinning these regional differences remain unclear, further investigations are imperative to elucidate the intricate processes involved.

Due to the cardiac toxicity associated with KCNH2 inhibitors that may lead to long QT syndrome, we propose several strategies to address this challenge. Firstly, we suggest modifying the chemical structure of known KCNH2 inhibitors to render them non-absorbable in circulation. This would allow for their targeted action solely within the intestine, akin to the mechanism of action of acarbose, a commonly used hypoglycemic drug.^47^ Secondly, we aim to conduct a comprehensive analysis of the structural variances among KCNH2 potassium channels in different organs (including heart, pancreatic islets, and intestines), utilizing techniques such as cryo-electron microscopy. Our goal is to identify KCNH2 blockers that selectively target intestinal KCNH2 while minimizing effects on cardiac KCNH2 channels, thus reducing the risk of cardiac toxicity. We draw inspiration from the example of insulin-tropic sulfonylureas--glimepiride, which selectively binds to pancreatic SUR1 receptors, avoiding adverse effects on cardiovascular K_ATP_ channels.^48,49^ By pursuing these strategies, we can harness the therapeutic potential of KCNH2 inhibition in the management of metabolic diseases while mitigating associated safety concerns, ultimately offering new avenues for effective and safe treatment options.

In conclusion, our study demonstrates the crucial role of the KCNH2 channel in regulating incretin secretion from EECs. Considering the pivotal role of incretins in appetite control and glucose metabolism, KCNH2 emerges as a promising novel target for the treatment of obesity and diabetes. Therefore, future drug development efforts should focus on compounds that selectively target intestinal KCNH2 without entering the circulation. Given the expression of various Kv channels in enteroendocrine cells,^17^ further exploration of the roles of additional Kv channels in regulating incretins is essential for a comprehensive understanding of enteroendocrine cell secretion mechanisms.

## Materials and methods

For further information regarding the data provided in this section, please refer to the Supplementary method as well.

### Animals

KCNH2^fl/fl^ mice and Vil1-iCre mice were constructed using CRISPR/Cas9 technology by GemPharmatech Co., Ltd (Nanjing, China) from C57BL/6 background mice. KCNH2 sgRNA direct Cas9 endonuclease cleavage in upstream of exon 1 and intron 9–10, and create a DSB (double-strand break). Following the repair of these breaks, homologous recombination will introduce LoxP sites upstream of exon 1 and intron 9–10, respectively, resulting in the construction of KCNH2^fl/fl^ mice. KCNH2^fl/fl^ mice were bred with Vil1-iCre mice to derive KCNH2 gut epithelial cell-conditional knock-out (KCNH2 CKO) mice. The genotyping of the KCNH2 CKO mice was combined with the primers of KCNH2^fl/fl^ mice and Vil1-iCre mice. Mice phenotypic analyses were carried out in accordance with earlier instructions.^50^ KCNH2^fl/fl^ primers: Forward 5′-CAATAGCAGCTCCACTGTTCCACTT-3′ and Reverse 5′-GATGGCTCATCTAACCAGGGAATAGCT-3′, Vil1-iCre primers: Forward 5′-ATGCCCACCAAAGTCATCAGTGTAG-3′ and Reverse 5′-AGTTTCCAAACTCCAGGTGACAGG-3′. Mice were kept under a 12-h light/dark cycle, and maintained on regular-chow diet or high-fat diet (HFD) (comprising 60% fat, 20% carbohydrate, and 20% protein by caloric content; Research DIETS, D12492, New Brunswick, NJ, USA). All mice had unrestricted access to food and water. Age-matched 12–16 weeks old male mice were used for the experiments as indicated in the figure legends. Animal procedures adhered to the national ethical guidelines implemented by our Institutional Animal Care and Use Committee and were approved by the Ethical Review Committee of the Institute of Zoology, Capital Medical University, China.

### Cell culture and transfection

STC-1 cells were cultured in high-glucose Dulbecco’s modified Eagle medium (DMEM) containing 25 mM glucose, supplemented with 10% fetal bovine serum (FBS) and 1x Penicillin-Streptomycin solution.^51^ For transient expression studies, STC-1 cells were transfected with 50 nM NC siRNAs or siRNAs targeting mouse KCNH2 according to the manufacturer’s protocol. After 48 h of transfection, RNA isolation was conducted for real-time PCR analysis and secretion studies, or cells were reseeded for voltage clamp measurements and intracellular calcium concentration measurements.

### Tissue histology and immunofluorescence

The duodenum (the top 10 cm of the small intestine) and ileum (the bottom 10 cm of the small intestine) were dissected from cervically dislocated C57BL/6 mice (12 weeks old). The full description of the immunofluorescence staining process and antibodies utilized are described in the Supplementary method and Supplementary Table 1. The microscopic images were obtained with 3D Histech Digital Pathology System and CaseViewer software for tissue sections and with an FV-3000 confocal laser scanning microscope and FV31S-SW software for STC-1 cells. At least three separate experiments are included in each image.

### Glucose and insulin tolerance tests

As described elsewhere, an oral glucose tolerance test (OGTT) or intraperitoneal glucose tolerance test (IPGTT) was performed on mice.^52^ The intraperitoneal insulin tolerance test (IPITT, 0.75 units of human insulin per kilogram of body weight) was conducted on mice. The figure legends indicate the specific glucose doses for each experiment. Blood glucose levels were measured by an automated glucometer, ACCU-CHEK® Performa (Roche). Approximately 50 μL of blood samples collected from the angular vein were mixed with an inhibitor (Aprotinin, Diprotin A, and Cocktail) to assay hormone concentrations during the OGTT. Serum GIP, GLP-1, insulin, PYY, cholecystokinin (CCK), somatostatin, dipeptidyl-peptidase IV (DPP-4), GLP-2 and glucagon concentrations were determined using an ELISA kit in accordance with the manufacturer’s instructions.

### Primary murine intestinal cultures

The intestines of mice aged 12–16 weeks were sacrificed by cervical dislocation and placed into ice-cold Leibovitz media. As previously stated, murine primary intestinal epithelial cell cultures were produced.^53^ In short, the intestine was opened lengthwise and washed in PBS after the muscular layers were removed, then it was sliced into 1–2 mm^2^ pieces. The tissue pieces were treated in 4 mmol/L EDTA cold chelation buffer for 30 min on ice. Tissue pieces were forcefully resuspended in cold 10% FBS in PBS using a 10 mL pipette. The resuspension/sedimentation process was usually repeated six to eight times, and isolated crypts were centrifuged at 200 × *g* and resuspended in DMEM (25 mM glucose) supplemented with 10% FBS, 2 mM glutamine, 1x Penicillin-Streptomycin solution. Twenty-four well plates covered with 1% v/v Matrigel were used to plate intestinal cell/crypt suspensions.

### Incretin secretion assay

An equal number of STC-1 cells were plated in six-well plates and transfected with scramble siRNAs or siRNAs targeting mouse KCNH2 for 48 h. Secretion investigations on primary intestinal cultures were carried at about 24 h after plating. Cells were rinsed with Hanks balanced salt solution (HBSS) and incubated in Krebs–Ringer bicarbonate buffer (KRBB) or KRBB supplemented with 10 mM EGTA for 30 min followed by the same buffer or 10 mM glucose or 10 mM glucose supplemented with 10 mM EGTA or 10 mM glucose supplemented with 10 μM Forskolin plus 10 μM IBMX (F/I) or 50 μM α-LA for additional 2 h.^33,34,39^ In another group of experiments, STC-1 cells were incubated in KRBB for 30 min followed by 10 mM glucose KRBB plus vehicle (DMSO) or 10 mM glucose KRBB supplemented with 10 μM Dofetilide for an additional 2 h.^54^ The cells were lysed using lysis buffer and the media were collected after a 2-h incubation. The supernatant collected from STC-1 cells was concentrated by Amicon Ultra 30 K. Incretins in the supernatant and lysates were measured using GLP-1, GIP, GLP-2 and glucagon ELISA kit, and an Infinite 200 Pro Reader. The amount of incretin, GLP-2, and glucagon secreted was normalized by incretin, GLP-2 and glucagon content remaining in the cells, respectively.

### Western blot

Western blot experiments were carried out as previously described.^55^ Tissues and cells were lysed in the lysis solution. The concentration of protein in the lysates was measured using a BCA protein assay kit. A total of 30 μg of total protein was placed onto polyacrylamide gels that were precast ranging from 7.5 to 12%. The enhanced chemiluminescence and an LAS-500 chemiluminescence detection system was used to detect the immunoreactive signal. Optical density values of immunoreactive bands were calculated using Image J software. The detailed WB protocols and antibodies utilized are provided in in the Supplementary Method and Supplementary Table 1.

### qRT-PCR

In our study, standard techniques were employed for mRNA isolation and qPCR, with detailed experimental procedures provided in the Supplementary method. Triplicate reactions were conducted for each sample. Primer sequences used for the qRT-PCR be found in Supplementary Table 2.

### Patch-clamp experiments

The STC-1 cells were first transfected with siRNA and plated in 3.5 cm dishes and cultured for more than 24 h before experiments. As previously described, experiments were carried out on single cell.^36,55^ Kv currents were recorded by clamping the membrane potential of STC-1 cells at −70 mV for 0.5 s, followed by a series of depolarizing stimuli of −70 to +70 mV for 2 s, with a step of 10 mV, and finally returning to the resting potential of −70 mV for 0.5 s. STC-1 cells were exposed to a 0.5 nA current injection for 50 ms in order to induce action potentials. The standard pipette solution and the standard bath solution utilized are provided in the Supplementary Method. Test chemicals were applied straight to the recording chamber while studies were conducted at room temperature.

### Calcium imaging in STC-1 cells

STC-1 cells were seeded on glass coverslips for 24 h, rinsed with KRBB (0 mM glucose), incubated in KRBB (0 mM glucose) containing 2 μM Fura-4-AM (Dojindo, Japan) for 30 min at 37 °C. Subsequently, cells were washed twice using KRBB without Fluo4-AM and pre-incubated 0 mM glucose KRBB for an additional half-hour and then stimulated with 10 mM glucose KRBB supplemented with 10 μM Forskolin plus 10 μM IBMX or 10 mM glucose KRBB supplemented with 10 μM dofetilide or vehicle. A Delta Vision Ultra High-Resolution Microscope (GE, USA) equipped with a 60x objective was used for the experiments. Sequential confocal images of cells were captured for a total of 31 min, beginning from 60 s prior to stimulation at 5 s internals, and analyzed using an Image J software. The Ca^2+^ concentrations were calculated as follows: F/F0 = (F - Base)/(F0 - Base) (F = real-time fluorescence measurement, F0 = the average baseline fluorescence density in the first 30 s prior to stimulation, Base = Background fluorescence).

### Flow cytometry

We constructed GLU-Venus transgenic mice (expressing the Venus fluorescent dye specifically in cells producing proglucagon).^29,56^ We isolated primary intestinal epithelial cells from the duodenum and ileum, respectively, and used fluorescence-activated cell sorting (FACS) to isolate mouse Venus-positive cells.^22,23,57^ As previously stated, intestinal tissues were cut into 1–2 mm^2^ segments and dissociated into individual cells using 5 mmol/L EDTA in calcium-free HBSS. Subsequently, single-cell suspensions were isolated by flow cytometry using FACS AriaIII (BD, USA). By using gating based on side scatter, forward scatter, and pulse width, fragments and aggregates were eliminated. The purity of Venus-positive cells was approximately 95%, whereas negative (control) cells consisted mainly of other epithelial cell types. Up to 40,000 cells were sorted and then lysed in Trizol reagent for RNA extraction.

### Cell viability assay

STC-1 cells were incubated with different concentrations (1, 3, 10, 30, or 100 μM) of dofetilide for 2 h, respectively. The 3-(4,5-dimethylthiazol-2-yl)-2,5-diphenyltetra-zolium bromide (MTT) test was used to evaluate the effects of dofetilide on cell viability and cytotoxicity.

### Statistical analysis

All statistical analyses were performed with the software GraphPad Prism version 8.0 Data are provided as means ± SEM. Statistical significance was verified using unpaired Student’s *t*-test or one-way ANOVA with a Tukey’s test. A *p* value of <0.05 was judged significant.

### Supplementary information

Supplementary_Materials-R3

## Data Availability

The data described in this study are available upon request from the corresponding author. We used the following previously released data sets: (1) Haber et al.^19^ NCBI Gene Expression Omnibus ID GSE92332. A single-cell analysis of the small intestinal epithelium. https://www.ncbi.nlm.nih.gov/geo/query/acc.cgi?acc=GSE92332. (2) Gehart et al.^20^ NCBI Gene Expression Omnibus ID GSE113561. Identification of enteroendocrine regulators by real-time single-cell differentiation mapping. https://www.ncbi.nlm.nih.gov/geo/query/acc.cgi?acc=GSE113561.
