# Supplementary material for: Potassium voltage-gated channel subfamily H member 2 (KCNH2) is a promising target for incretin secretagogue therapies
Source: Signal Transduct Target Ther. 2024 Aug 12;9:207. doi: 10.1038/s41392-024-01923-z (PMC11317495; doi:10.1038/s41392-024-01923-z)
Supplement: Supplementary file 1 — Supplementary_Materials-R3 [file 41392_2024_1923_MOESM1_ESM.docx]

**Supplementary Materials for**

**Potassium voltage-gated channel subfamily H member 2 (KCNH2) is a promising target for incretin secretagogue therapies**

Running title: KCNH2 for incretin secretagogue therapies

Ying-Chao Yuan^1,2*^, Hao Wang^1,2*🖂^, Ze-Ju Jiang^1,2^, Chang Liu^1^, Qi Li^1,2^, Si-Rui Zhou^1,2^, and Jin-Kui Yang^1,2,🖂^

Correspondence to: jkyang@ccmu.edu.cn (Jin-Kui Yang) or hwang@mail.ccmu.edu.cn (Hao Wang)

**This PDF file includes:**

Materials and Methods

Supplementary Figures S1 to S8

Supplementary Tables S1 to S3

**Materials and methods**

**Cell culture and transfection**

STC-1 cells were cultured in high-glucose Dulbecco's modified Eagle medium (DMEM) containing 25 mM glucose. 10% fetal bovine serum (FBS) and 1x Penicillin-Streptomycin solution were added to the culture medium. The cells were cultivated in a humidified incubator at 37°C with 95% air and 5% CO_2_. For transient expression studies, STC-1 cells at 70% confluence were transfected with 50 nM NC siRNAs (sc-37007, Santa Cruz, USA) or siRNAs targeting mouse KCNH2 (sc-42498, Santa Cruz, USA) using Lipofectamine RNAiMAX (Invitrogen) according to the manufacturer’s protocol. After 48 hours of transfection, RNA isolation was conducted for real-time PCR analysis and secretion studies, or cells were reseeded for voltage clamp measurements and intracellular calcium concentration measurements.

**Tissue histology and immunofluorescence**

The duodenum and ileum tissues were fixed in 4% paraformaldehyde (PFA) for 24-48 hours, cryoprotected in 30% sucrose solution (w/v in phosphate-buffered saline (PBS)), and sectioned into 10 μm slices for H&E staining. After fixing STC-1 cells and murine frozen sections for 30 minutes at room temperature using 4% paraformaldehyde in PBS, PBS washed three times, and 0.1% Triton X-100 was permeabilized for 30 minutes in PBS. For 30 minutes, blocking was done using PBS containing 1% bovine serum albumin (BSA). After an overnight incubation period at 4°C with the primary antibody, the coverslips were rinsed three times with PBS and incubated for another hour at room temperature with Alexa Fluor 488- or 568-conjugated secondary antibody (Invitrogen, USA). After being cleaned in PBS five times, the samples were affixed using a mounting solution containing DAPI (utilized for nuclear staining) reagent (Beyotime, Shanghai, China). The microscopic images were obtained with 3D Histech Digital Pathology System and CaseViewer software for tissue sections and with an FV-3000 confocal laser scanning microscope (Olympus) outfitted with a 100 × oil immersion objective lens (1.45 NA) and FV31S-SW software for STC-1 cells. At least three separate experiments are included in each image.

**Primary murine intestinal cultures**

In short, the intestine was opened lengthwise and washed in PBS after the muscular layers were removed, then it was sliced into 1-2 mm^2^ pieces. Duodenal cultures included tissue from the upper ten centimeters of the small intestine, located distal to the stomach, and ileal cultures contained tissue from the lower 10 cm of the small intestine, proximal to the caecum. The tissue pieces were treated in 4 mmol/L EDTA cold chelation buffer for 30 minutes on ice. To separate intestinal crypts, tissue pieces were forcefully resuspended in cold 10% FBS in PBS using a 10 mL pipette after the EDTA buffer was removed. After allowing the tissue fragments to settle for a minute under normal gravity, the supernatant was taken out and examined using inverted microscopy. The resuspension/sedimentation process was usually repeated 6-8 times, and isolated crypts were centrifuged at 200× g and resuspended in DMEM (25 mM glucose) supplemented with 10% FBS, 2 mM glutamine, 1x Penicillin-Streptomycin solution. 24-well plates covered with 1% v/v Matrigel (BD Bioscience, Oxford, UK) were used to plate intestinal cell/crypt suspensions, which were then incubated for the whole night at 37°C in 5% CO2.

**Western blot**

Western blot experiments were carried out as previously described. Tissues and cells were lysed in lysis solution that contained 20 mM Tris-HCl pH 7.5, 150 mM NaCl, 1 mM MgCl_2_, 10 mM EGTA, 1% Triton X-100, 1 mM PMSF, and complete protease inhibitor cocktail (Roche). Following a 10-minute centrifugation at 14,000 rpm at 4°C, the supernatants were gathered as protein. Cell lysed proteins separated by SDS-PAGE were transferred to a polyvinylidene difluoride membrane (Millipore). After blocking the membranes with TBST (TBS plus 0.1% Tween-20) containing 5% nonfat dry milk powder, the membranes were incubated with primary antibody overnight at 4°C. After three TBST rinses, the membranes were incubated with horseradish peroxidase-conjugated secondary antibody (1:2000, Beyotime, China) in TBST containing 5% nonfat dried milk powder for 1 h at room temperature. This was followed by five TBST rinses. Next, the enhanced chemiluminescence (Amersham Biosciences, USA) and an LAS-500 chemiluminescence detection system (GE Healthcare Bioscience, USA) was used to detected the immunoreactive signal. Optical density values of immunoreactive bands were calculated using Image J software. Supplementary Table 1 lists the sources of antibodies and the concentrations at which they were employed.

**qRT-PCR**

RNA extraction was performed using Trizol Reagent (Thermo Fisher Scientific, USA) according to the manufacturer's instructions. Next, using SuperScriptIII Reverse Transcriptase (Thermo Fisher Scientific) to synthesize the first-strand cDNA from RNA template. Subsequently, quantitative PCR experiments were conducted using cDNA template, particular PCR primers and TransStart Tip Green qPCR SuperMix (TransGen Biotech, Beijing, China). Triplicate reactions were conducted for each sample. 36B4 acted as the housekeeping gene to normalize the expression of other genes. Primer sequences used for the qRT-PCR be found in Supplementary Table 2.

**Microbiome analysis**

Fresh fecal samples were obtained from mice in the morning during ad libitum feeding. Frozen fecal samples were delivered to Novogene Bioinformatics Ltd. and a fecal DNA kit (TianGen) was used to extract DNA according to the manufacturer's recommendations. The 16S rRNA genes of different regions (16SV3-V4) were amplified using specific primers (515F-806R) with barcodes. All PCR reactions were performed with 15 µL Phusion® High Fidelity PCR Master Mix (New England Biolabs). The Universal DNA Purification Kit (TianGen) was used to purify the combined PCR results. Using the NEB Next®Ultra^TM^ II FS DNA PCR-free Library Prep Kit (New England Biolabs), sequencing libraries were created and indexed. The libraries were quantified using Qubit and real-time PCR. Quantitative libraries were assembled and sequenced on the Illumina platform based on the concentration of validated libraries and the amount of data required. The sequences of primers and barcodes were cut to truncate pair-end reads, which were then merged using FLASH (http://ccb.jhu.edu/software/FLASH/). Fastp software was used to perform quality filtering of raw tags, resulting in high-quality clean tags. The UCHIME algorithm (http://www.drive5.com/usearch/manual/uchime_algorithm.html) was used to compare the tags with a reference database (Silva database, https://www.arb-silva.de/) in order to identify and eliminate chimeric sequences and then legitimate tags were acquired. To acquire the initial ASVs, noise reduction was carried out using the DADA2 or deblur module in the QIIME2 program (Version QIIME2-202202), which was used to annotate species. The data from each sample were then homogenized, with the homogenization standard being the data with the fewest data points in the sample. The homogenized data served as the foundation for the next Alpha and Beta diversity analyses.

**Patch-clamp experiments**

Electrodes were fire-polished with a microforge (P-1000; Sutter). When filled with pipette solution, the resistance of the electrodes ranged between 4-6 MΩ. An EPC-10 amplifier and PULSE software (HEKA Electronik, Lambrecht, Germany) were used to patch-clamp STC-1 cells throughout the whole cell. Data were analyzed by Clampfit software (Axon Instruments). Before study, the cells washed with standard extracellular solution and media were replaced with 1 ml the standard extracellular solution. Kv currents were recorded by clamping the membrane potential of STC-1 cells at -70 mV for 0.5 s, followed by a series of depolarizing stimuli of -70 - +70 mV for 2 s, with a step of 10 mV, and finally returning to the resting potential of -70 mV for 0.5 s. The intracellular solutions contained (mmol/L): 130 KCl, 10 NaCl, 10 HEPES, 1 EGTA, 2 MgC1_2_, 0.5 CaC1_2_, adjust pH to 7.3 with KOH and extracellular solutions contained (mmol/L): 140 NaCl, 10 Glucose, 4 KCl, 10 HEPES, 1 CaCl_2_, 2 MgC1_2_, 1 KH_2_PO_4_, adjust pH to 7.3 with NaOH. STC-1 cells were exposed to a 0.5 nA current injection for 50 ms in order to induce action potentials. For recording action potentials, the standard pipette solution comprised (mmol/L): 76 K_2_SO_4_, 10 KCl, 10 NaCl, 55 sucrose, 1 MgCl_2_ and 10 HEPES (pH 7.2 with KOH) and the standard bath solution contained (mmol/L): 5.6 KCl, 138 NaCl, 2.6 CaCl_2_, 1.2 MgCl_2_, 1 Glucose and 10 HEPES (pH 7.4 with NaOH). Test chemicals were applied straight to the recording chamber while studies were conducted at room temperature.

**Monitoring of food and water intake**

The Comprehensive Laboratory Animal Monitoring System (CLAMS, Columbus Instruments at McMaster) was used to track the intake of food and water. High-fat-fed mice were experimented on at 19 weeks. The mice were first acclimated in the device for 24 hours before data recording began. The experiment started at 8:30 am and lasted for 5 days. During the experiment, food and water intake data were collected every hour.

**scRNA-seq analyses**

Using a single cell RNA sequencing (scRNA-seq) dataset that is available to the public, we examined intestinal cells from mice. This dataset came from the NCBI Gene Expression Omnibus database (GSE92332) and was created by Haber et al. We used the RNA-SEQ dataset from Gehart et al to analyze mouse EECs (GSE113561). The the Python package scanpy (1.9.0) was used to perform quality control, normalization, standardization, feature selection, and dimensionality reduction of the scRNA-seq data matrix. Cell selection was based on gene expression levels, gene expression counts, and the ratio of mitochondrial gene expression. After normalization, 2000 highly variable genes were selected based on the dispersion of gene expression. These 2000 genes were then used for principal component analysis to reduce dimensionality to 30. These 30 features were utilized to create a k-nearest neighbor graph, and the Leiden algorithm was used to cluster the cells. The clusters of cells were then classified according to their previously annotated cell types.


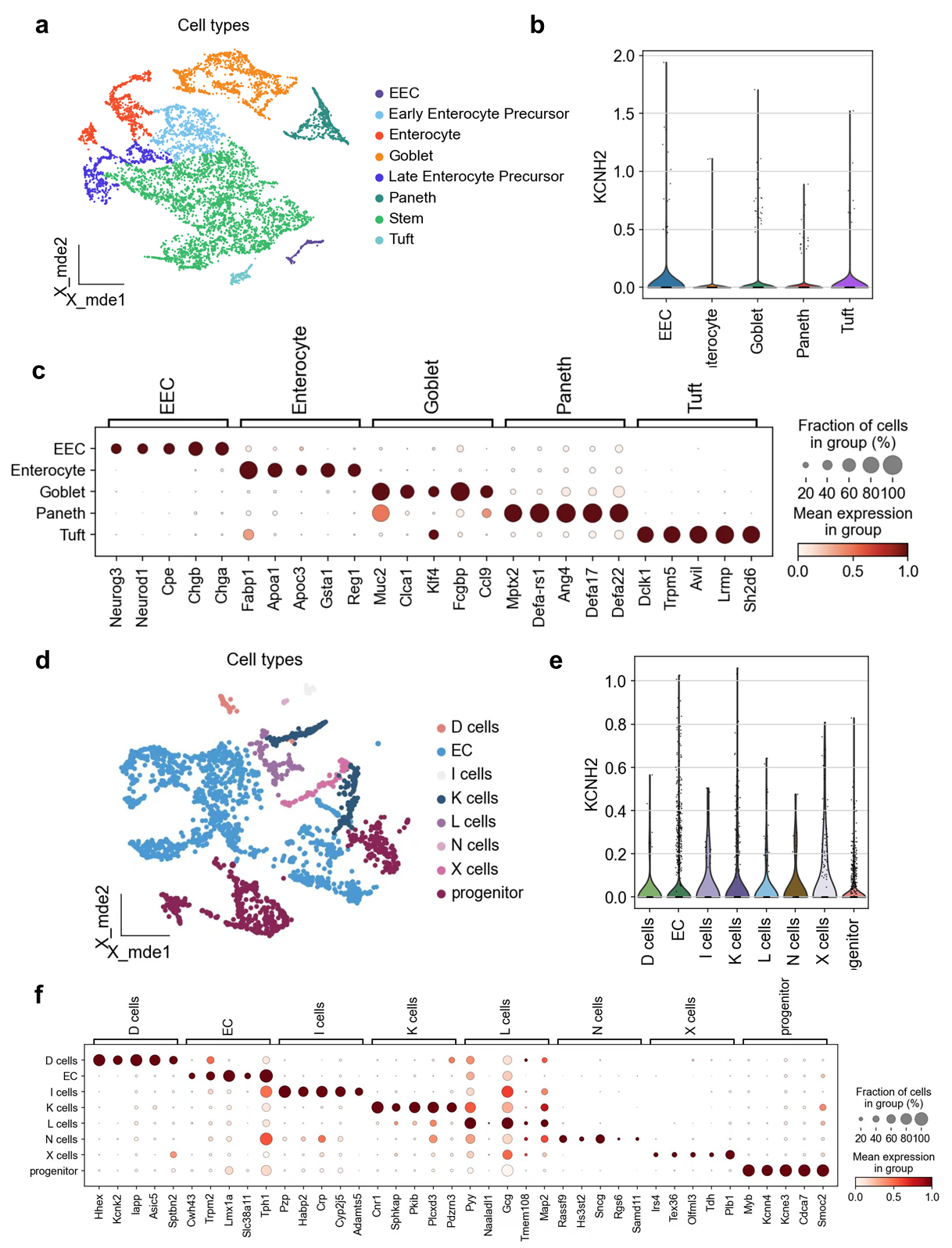


**Supplementary Fig. 1** Mature intestinal epithelial cells, particularly EECs, express KCNH2 in public single-cell datasets from mouse. **a-c** An analysis was conducted on the mouse intestinal epithelial cell single-cell transcriptome dataset (GSE92332) to determine the expression of KCNH2 in each epithelial cell subtype. The Uniform Manifold Approximation and Projection (UMAP) plot of the mouse intestinal epithelial cell subtype transcriptome data shows 10 cell clusters (**a**). Expression of the KCNH2 gene in different intestinal epithelial cells subtype is shown (**b**). The normalized expression of enriched feature genes in different intestinal epithelial cells subtype is shown (**c**). **d-f** Single-cell transcriptional dataset (GSE113561) of mouse enteroendocrine cells was analyzed to determine the expression of each enteroendocrine cell subtype that expresses KCNH2. The UMAP plot of the mouse EECs transcriptome data shows 8 cell clusters (**d**). Expression of the KCNH2 in different EECs subtype is shown (**e**). The normalized expression of enriched feature genes in different EECs subtype is shown (**f**).

**
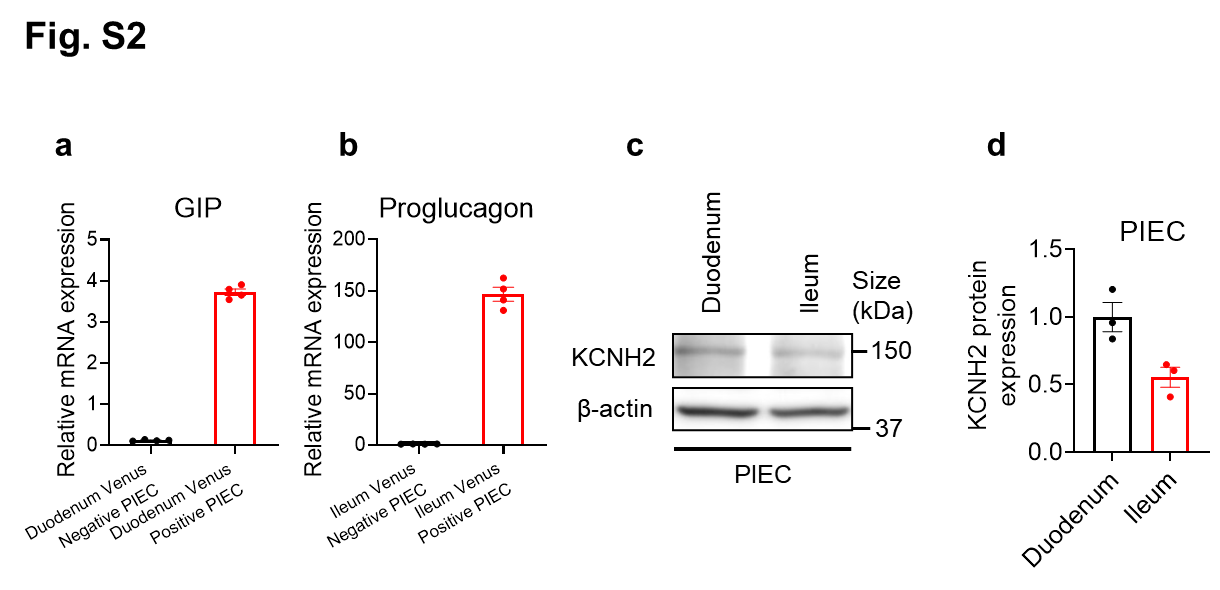
**

**Supplementary Fig. 2** Protein levels of KCNH2 in primary intestinal epithelial cells. **a** Relative expression of GIP in murine duodenum Venus-positive primary intestinal epithelial cells (PIEC) and duodenum Venus-negative PIEC by qRT-PCR (n = 4 replicates for each group). Gene expression was calculated as 2^-ΔΔct. 36B4 was used as an internal control. **b** Relative expression of proglucagon in murine ileum Venus-positive PIEC and Venus-negative PIEC by qRT-PCR (n = 4 replicates for each group). **c, d** KCNH2 protein levels in murine PIEC from the duodenum and ileum (n = 3 mice per group).

**
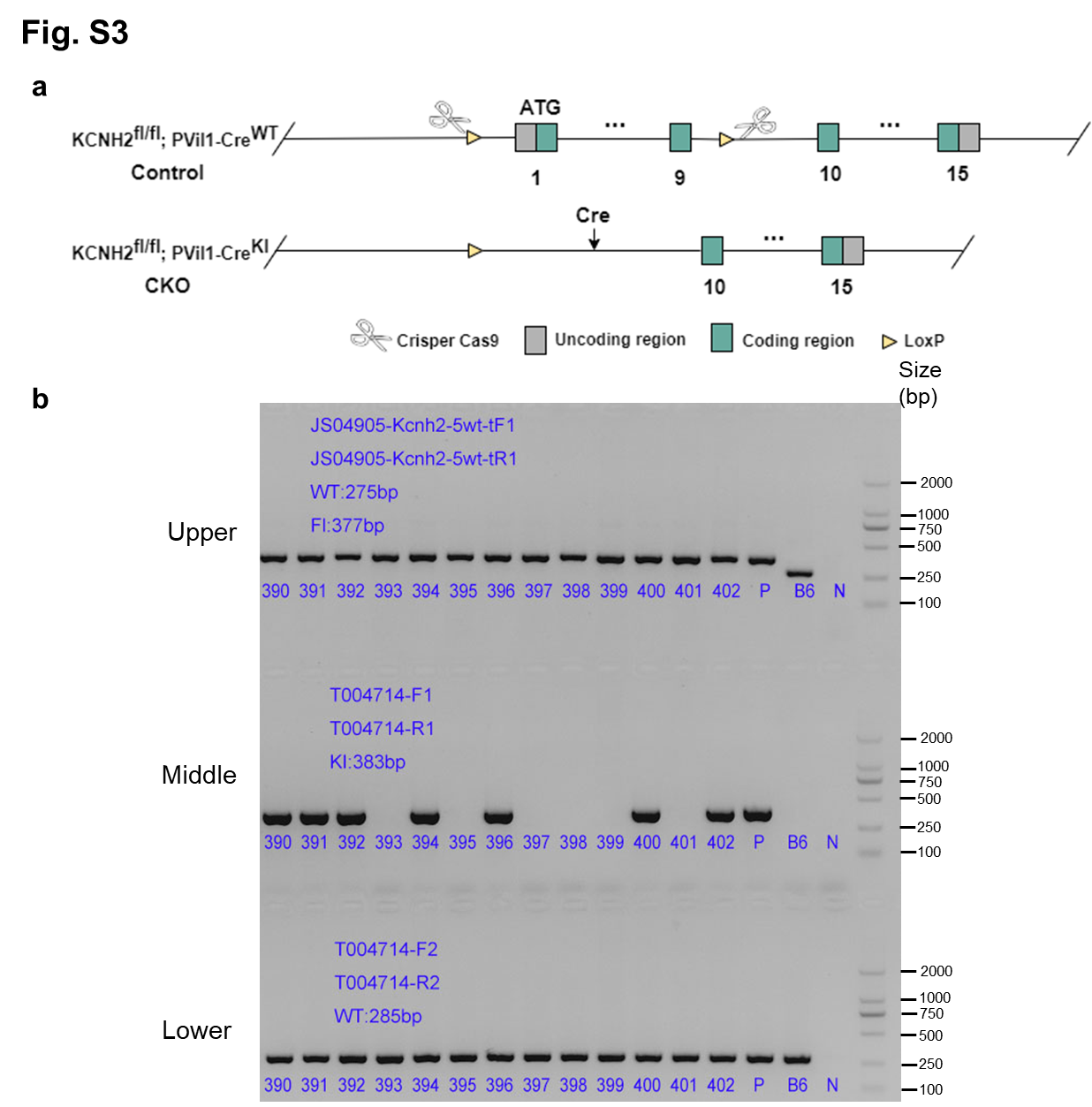
**

**Supplementary Fig. 3** Construction and identification of KCNH2 gut epithelial cell conditional knockout mice (CKO). **a** Construction of KCNH2 CKO mice. KCNH2 sgRNA direct Cas9 endonuclease cleavage in upstream of exon 1 and intron 9-10, and create a DSB (double-strand break). Such breaks will be repaired, and result in LoxP sites inserted into upstream of exon 1 and intron 9-10 respectively by homologous recombination, resulting in the construction of KCNH2^fl/fl^ mice. KCNH2^fl/fl^ mice were crossed with Vil1-iCre mice to derive KCNH2 gut epithelial cell conditional knock out (KCNH2 CKO) mice. **b** Identification of KCNH2 CKO mice by Gel Electrophoresis. The results indicate that mice whose number are 390, 391, 392, 394, 396, 400, and 402 correspond to KCNH2^fl/fl^ Vil1-iCre^KI^, referred to as KCNH2 CKO mice. Mice whose number are 393, 395, 397, 398, 399, and 401 correspond to KCNH2^fl/fl^ Vil1-Cre^WT^, referred to as Control mice. Upper: Mice with the flox fragment display a band at 377 bp. Middle: Mice with the iCre^KI^ fragment display a band at 383 bp. Lower: Mice with the iCre^WT^ fragment display a band at 285 bp.

**
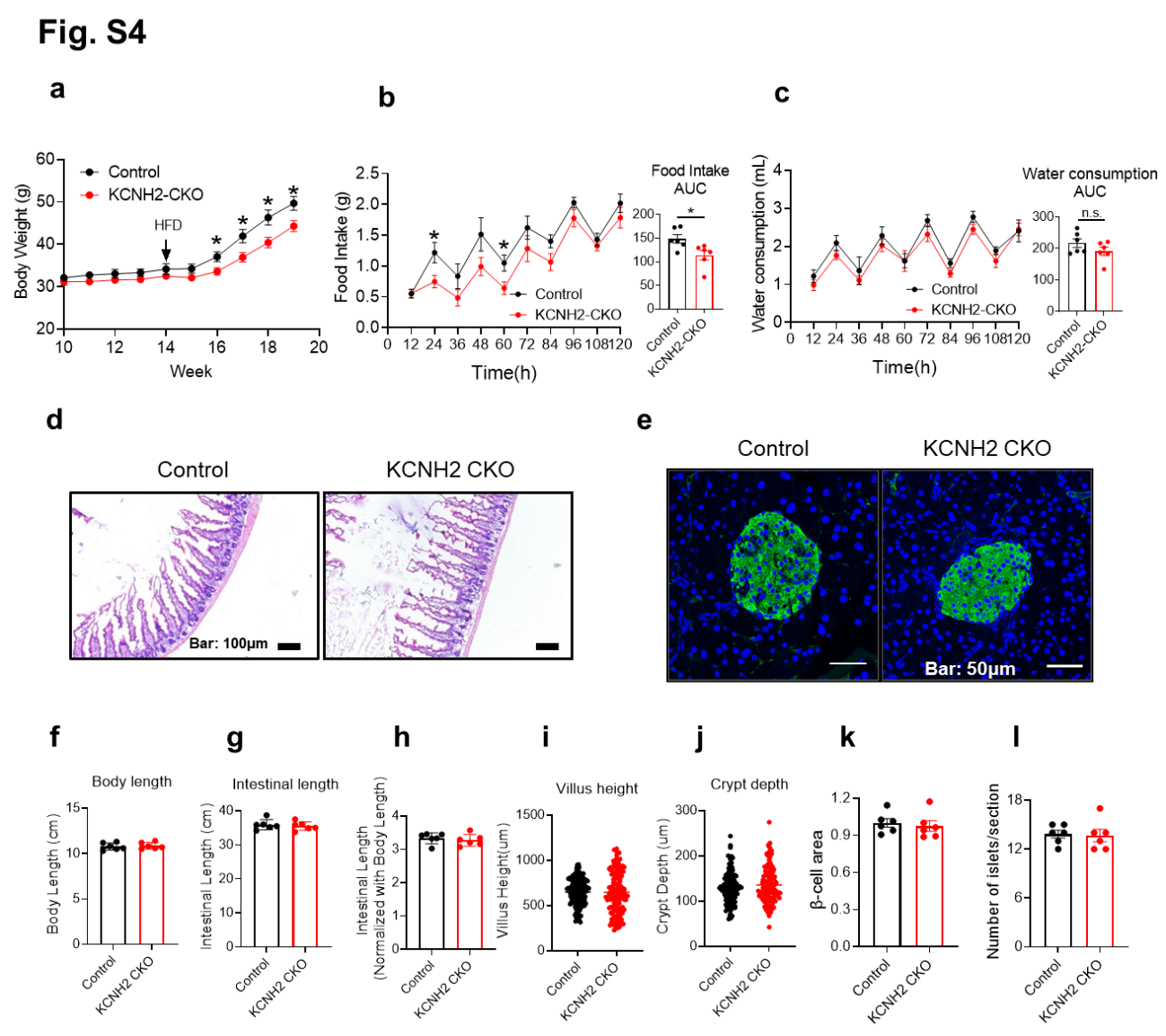
**

**Supplementary Fig. 4** KCNH2^fl/fl^ Vil1-iCre mice had no effect on growth of intestine and islets. **a** Body weight Body weights of KCNH2^fl/fl^ (Control, n=6) and KCNH2^fl/fl^; pVillin-Cre CKO, n=7) mice from 10 weeks to 19 weeks. Mice were started on a high-fat diet at week 15. **b** Food intake per 12 hours over 5 days in control and CKO mice and their AUC (n = 6 mice for each group). **c** Water consumption per 12 hours over 5 days in control and CKO mice and their AUC (n = 6 mice for each group). **d** H&E staining showing the histology of murine intestine (Control and CKO) , with a scale bar representing 100 μm. **e** Immunostaining of murine pancreas (Control and CKO) using insulin antibody (n = 4 mice for each group), with a scale bar representing 50 μm. **f-j** Body length (**f**), intestinal length (**g**), intestinal length (normalized with body length) (**h**) for Control and CKO mice (n = 6 mice for each group). **i-j** Villus height (**i**, Control, n = 139; CKO, n = 145, from 6 murine samples were analyzed) and crypt depth (**j**, Control, n = 126; CKO, n = 121, from 6 murine samples were analyzed) were quantified. **k-l** β-cell area (**k**) and number of islets/section (**l**) were quantified (n = 6 mice for each group). The values are presented as means ± SEM. The statistical significance of differences between means was assessed by the Student’s t test. *p < 0.05.

**
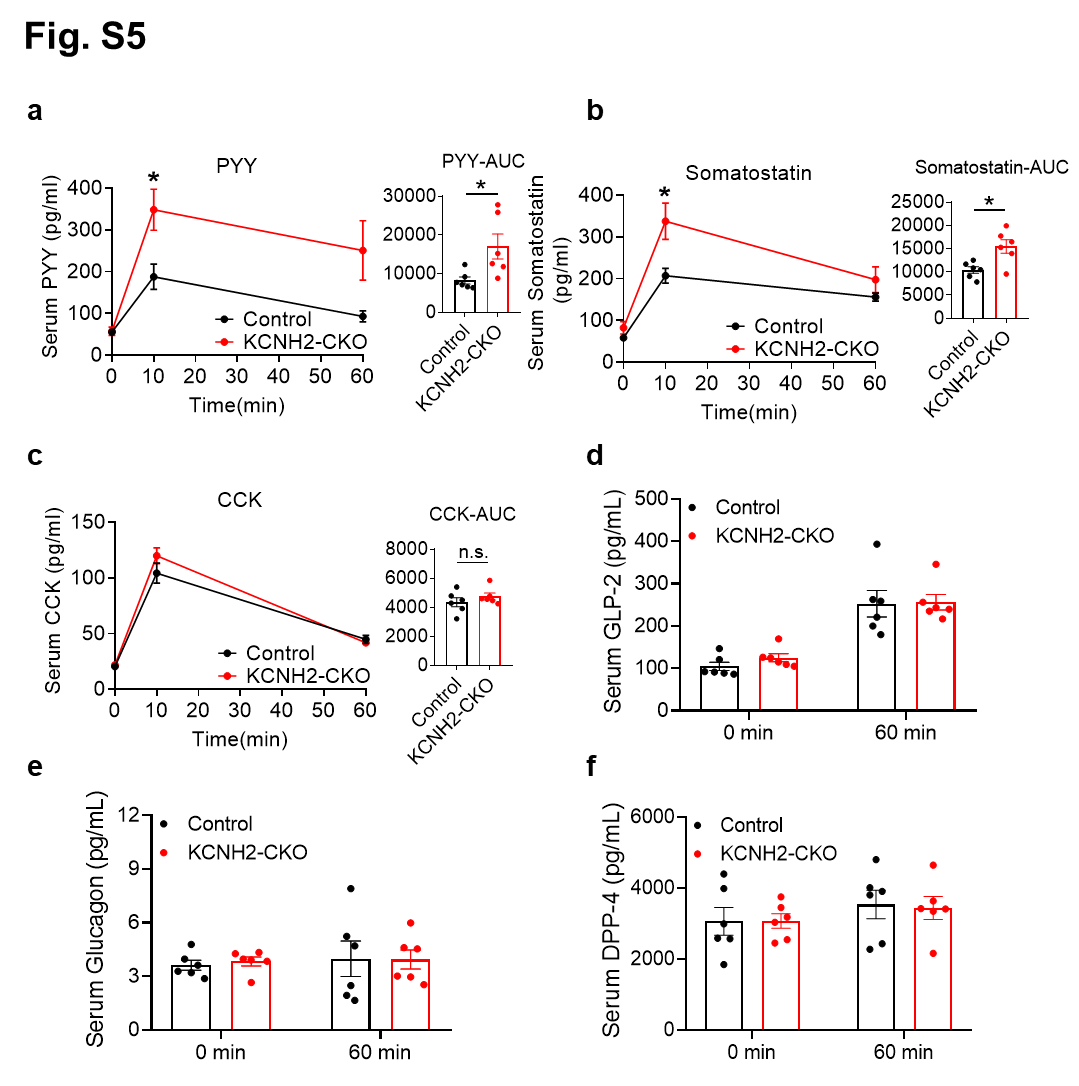
**

**Supplementary Fig. 5** KCNH2 deficiency enhances PYY and somatostatin secretion in response to glucose in vivo. **a-c** Serum PYY (**a**), somatostatin (**b**), and CCK levels (**c**), along with their AUC during OGTT (5 g/kg) for mice (HFD-fed for 6-8 weeks, started at week 8) (Control, n = 6; CKO, n = 6). **d-f** Serum GLP-2 (**d**), glucagon(**e**) and DPP-4 (**f**) levels at 0 min and 60 min after oral glucose load (5 g/kg) in mice (HFD-fed for 6-8 weeks, started at week 8) (Control, n = 6; CKO, n = 6).

**
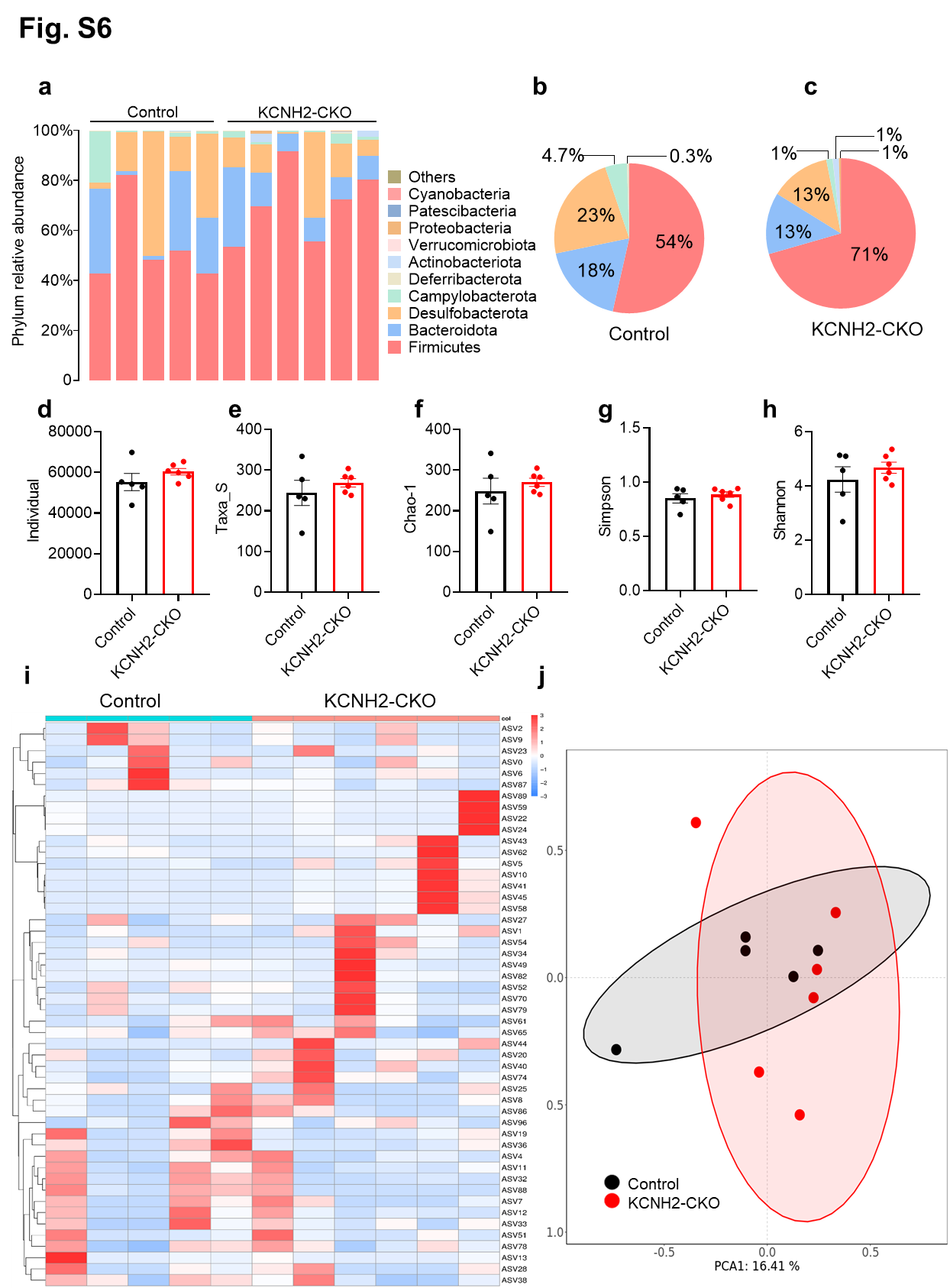
**

**Supplementary Fig. 6** Microbiota analysis of KCNH2^fl/fl^ Vil1-iCre mice and Control mice. **a-c** Stacked bar chart (**a**) and pie chart (**b-c**) showing the relative abundance of phyla in feces of HFD Control and CKO mice (HFD-fed started at week 8 for 6-8 weeks, Control, n = 5; CKO, n = 6). **d-h** Sample richness (**d**, individual; **e**, Taxa_S, detected richness; **f**, Chao-1, predicted richness) and alpha-diversity (**g**, Simpson; **h**, Shannon) were not significantly changed (Control, n = 5; CKO, n = 6). **i** Heatmap showing the relationship of fecal samples from Control and CKO mice. Top-50 differentially abundant OTUs (rows) and samples (columns) were arranged (Control, n = 5; CKO, n = 6). **j** Principal component analysis (PCA) of microbiota (Control, n = 5; CKO, n = 6). The values are presented as means ± SEM. The statistical significance of differences between means was assessed by the Student’s t test.


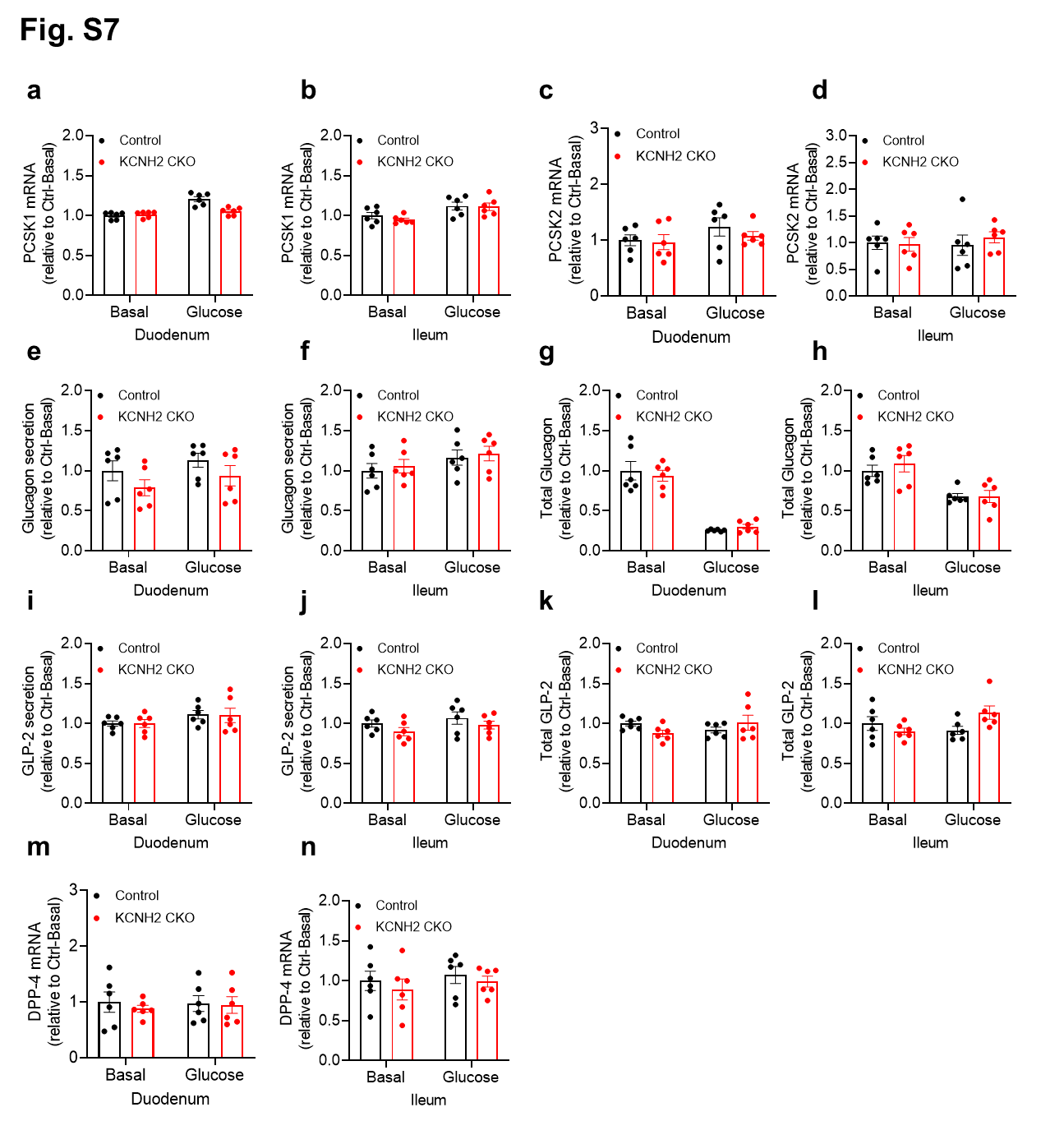


**Supplementary Fig. 7** KCNH2 knockout did not affect the expression of PCSK1/PCSK2 and DPP-4, the secretion and total amount of GLP-2 and glucagon. **a-b** PCSK1 mRNA expression in the PIEC of duodenum (**a**) and ileum (**b**) (n = 6 replicates for each group) by qRT-PCR. **c-d** PCSK2 mRNA expression in the PIEC of duodenum (**c**) and ileum (**d**) (n = 6 replicates for each group) by qRT-PCR. **e-f** In vitro basal conditions (0 mM glucose) and 10 mM glucose-induced glucagon secretion in duodenal (**e**) and ileal (**f**) PIEC of Control and CKO mice (n = 6 replicates per group). **g-h** total GIP content in duodenal (**g**) and ileal (**h**) PIEC of Control and CKO mice (n = 6 replicates per group). **i-j** In vitro basal conditions (0 mM glucose) and 10 mM glucose-induced GLP-2 secretion in duodenal (**i**) and ileal (**j**) PIEC of Control and CKO mice (n = 6 replicates per group). **k-l** Total GLP-2 content in duodenal (**k**) and ileal (**l**) PIEC of Control and CKO mice (n = 6 replicates per group). **m-n** DPP-4 mRNA expression in the PIEC of duodenum (**m**) and ileum (**n**) (n = 6 replicates for each group) by qRT-PCR. Gene expression was calculated as 2^-ΔΔct. 36B4 was used as an internal control. The values are presented as means ± SEM. The statistical significance of differences between means was assessed by the Student’s t test. *p < 0.05.


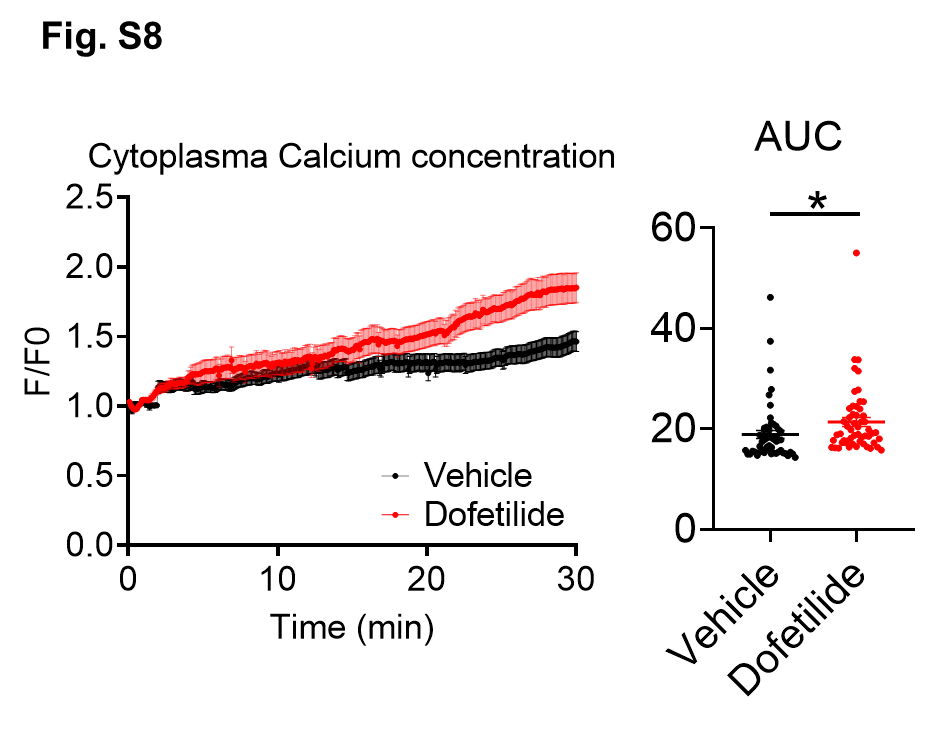


**Supplementary Fig. 8** Intracellular calcium concentration was determined using 2 μM Fluo 4-AM. Readings were taken every 5 seconds for a total of 31 minutes, with 60 seconds recorded prior to and 30 minutes following stimulation with 10 mM glucose KRBB supplemented with 10 μM dofetilide or vehicle. The fluorescence change ratio (F/F0) was recorded for STC-1 cells. The summary of area under curve (AUC) of STC-1 cells (Vehicle, n = 55; Dofetilide, n = 54).

**Supplementary Table 1 The sources of commercial reagents and the concentrations of the antibodies used for immunofluorescence (IF) and immunoblotting (IB).**

| **Antibody** | **Clone** | **Vendor** | **Catalog number** | **RRID** | **Dilution** | **Application** |
| --- | --- | --- | --- | --- | --- | --- |
| anti-KCNH2 | Rabbit polyclonal | Sigma-Aldrich, St.Louis, MO, USA | SAB2104244 | AB_10668591 | IB 1/1000; IF/100 | IB, IF |
| Insulin | Mouse monoclonal | Cell Signaling Technology, Inc., MA, USA | 8138S | AB_10949314 | 1/500 | IF |
| GIP | Rabbit polyclonal | Proteintech, Wuhan, China | 18034-1-AP | AB_2878484 | 1/200 | IF |
| GLP-1 | Mouse monoclonal | Abcam, Cambridge, UK | ab23472 | AB_447455 | 1/200 | IF |
| β-actin | Mouse monoclonal | Sigma-Aldrich, St.Louis, MO, USA | A5316 | AB_476743 | 1/10000 | IB |
| Rabbit IgG | Rabbit polyclonal | Proteintech, Wuhan, China | 30000-0-AP | AB_2819035 | 1/100 | IF |

**Supplemental Table 2 Primer for quantitative PCR**

| Target | Primer | Sequence |
| --- | --- | --- |
| GIP | Forward | CAGGTAGGAGGAGAAGACCTCAT |
|  | Reverse | CCTAGATTGTGTCCCCTAGCC |
| PYY | Forward | CCGCCATGGCCACAGTGCTAC |
|  | Reverse | CCCAGGGGCAGGGACATGGAGAA |
| GCG | Forward | GGCACATTCACCAGCGACTACA |
|  | Reverse | GCCCTCCAAGTAAGAACTCACATC |
| KCNH2 | Forward | ATGGCTCAGATCCAGGCAGTTA |
|  | Reverse | CAAGGAGAGCGGTCAGGTAATG |
| KCNH6 | Forward | CGTTTGTTGTCACACAGCTTCCTG |
|  | Reverse | TCCACAAAGCTGAGCGTGAACTG |
| KCNH7 | Forward | AACAGTGCAAATGACGCTGACGAC |
|  | Reverse | TCTTCACCATGGAGTCTGGTTGCT |
| PCSK1 | Forward | TCTGGTTGTCTGGACCTCTGAGT |
|  | Reverse | CATCAAGCCTGCCCCATTCTTT |
| PCSK2 | Forward | CATCACAGTCAACGCGACCAG |
|  | Reverse | TTTCTCAGGATACTTTGCAGG |
| DPP-4 | Forward | TTGTGGATAGCAAGCGAGTTG |
|  | Reverse | CACAGCTATTCCGCACTTGAA |
| Rplp0/36B4 | Forward | GGCCCTGCACTCTCGCTTTC |
|  | Reverse | TGCCAGGACGCGCTTGT |

**Supplementary Table 3 The sources of commercial reagents used in this paper**

| **Reagent** | **Source** | **Catalog number** |
| --- | --- | --- |
| DMEM | Gibco | C11995500BT |
| DMEM/F12 | Gibco | 11965092 |
| Fetal bovine serum | Gibco | 10099-141 |
| Lipofectamine 3000 | Invitrogen | L3000-015 |
| RNAiMAX | Invitrogen | 13778-150 |
| Trizol | Invitrogen | 15596018 |
| Superscript III | Invitrogen | 18080044 |
| TransStart Tip Green qPCR SuperMix | TransStart, Beijing, China | AQ141-04-P |
| Aprotinin | MACKLIN, Shanghai, China | A6353 |
| Diprotin A | MACKLIN, Shanghai, China | D873892 |
| Cocktail | Roche | 05056489001 |
| Leibovitz (L-15) | Invitrogen | 11415064 |
| BSA | Sigma | B2064 |
| Forskolin | Sigma | F6886 |
| IBMX | Sigma | I5879 |
| Fluo-4 AM | Dojindo, Tokyo, Japan | F312 |
| Dofetilide | MCE, USA | HY-B0232 |
| Multi Species GLP-1 Total ELISA kit | Millipore, Massachusetts, USA | EZGLP1T-36K |
| Rat/Mouse (Total) GIP ELISA kit | Millipore, Massachusetts, USA | EZRMGIP-55K |
| Rat/Mouse Insulin ELISA kit | Millipore, Massachusetts, USA | EZRMI-13K |
| mouse Glucagon ELISA kit | Crystal Chem, USA | 81518 |
| Mouse Dipeptidase 4 ELISA kit | Signalway Antibody, USA | EK2400 |
| Mouse Cck/cholecystokinin ELISA kit | Signalway Antibody, USA | EK19384 |
| Mouse somatostatin ELISA kit | Signalway Antibody, USA | EK1997 |
| Mouse peptide YY(PYY) ELISA kit | Signalway Antibody, USA | EK20650 |
| ELISA Kit for Glucagon Like Peptide 2 (GLP2) | Cloud-clone, Wuhan, China | CED059Mu |
